# Supplementary material for: Therapeutic role of miR-19a/b protection from influenza virus infection in patients with coronary heart disease
Source: Mol Ther Nucleic Acids. 2024 Feb 15;35(1):102149. doi: 10.1016/j.omtn.2024.102149 (PMC10907223; doi:10.1016/j.omtn.2024.102149)
Supplement: Document S1. Figures S1–S10 [file mmc1.pdf]

OMTN, Volume *35*

Supplemental information

Therapeutic role of miR-19a/b protection from influenza virus infection in patients with coronary heart disease

Yanan Xing, Lin Chen, Bin Hu, Yi Li, Huan Mai, Gaojian Li, Shuyi Han, Ye Wang, Yanyi Huang, Ying Tian, Wei Zhang, Yan Gao, and Hongxuan He

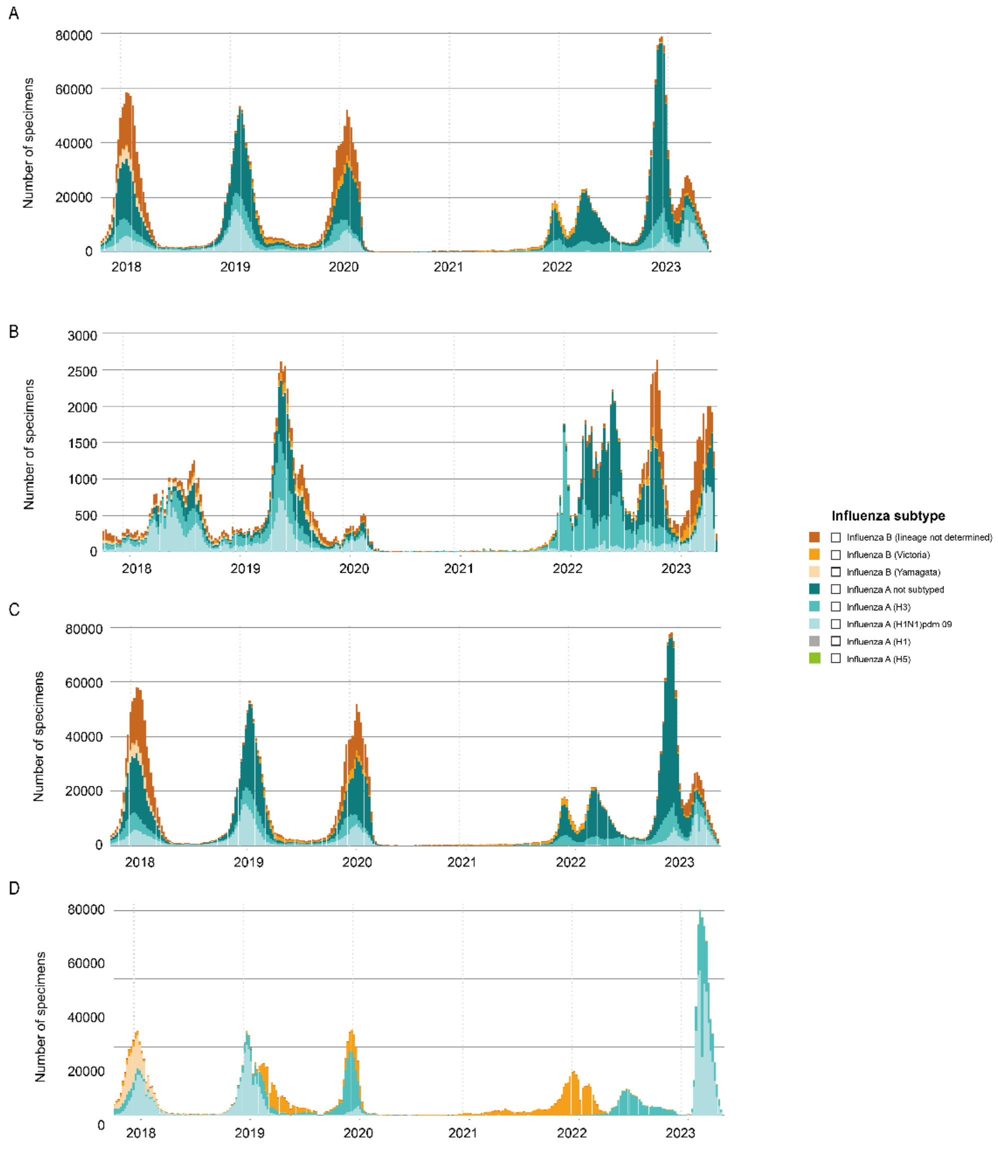

Figure S1 Patterns of influenza virus circulation in the global world、Northern (top) 、 Southern (bottom) Hemispheres and China, Jan 2018 to present. Data are based on laboratory confirmed influenza activity reported to WHO flunet. https://www.who.int/tools/flunet

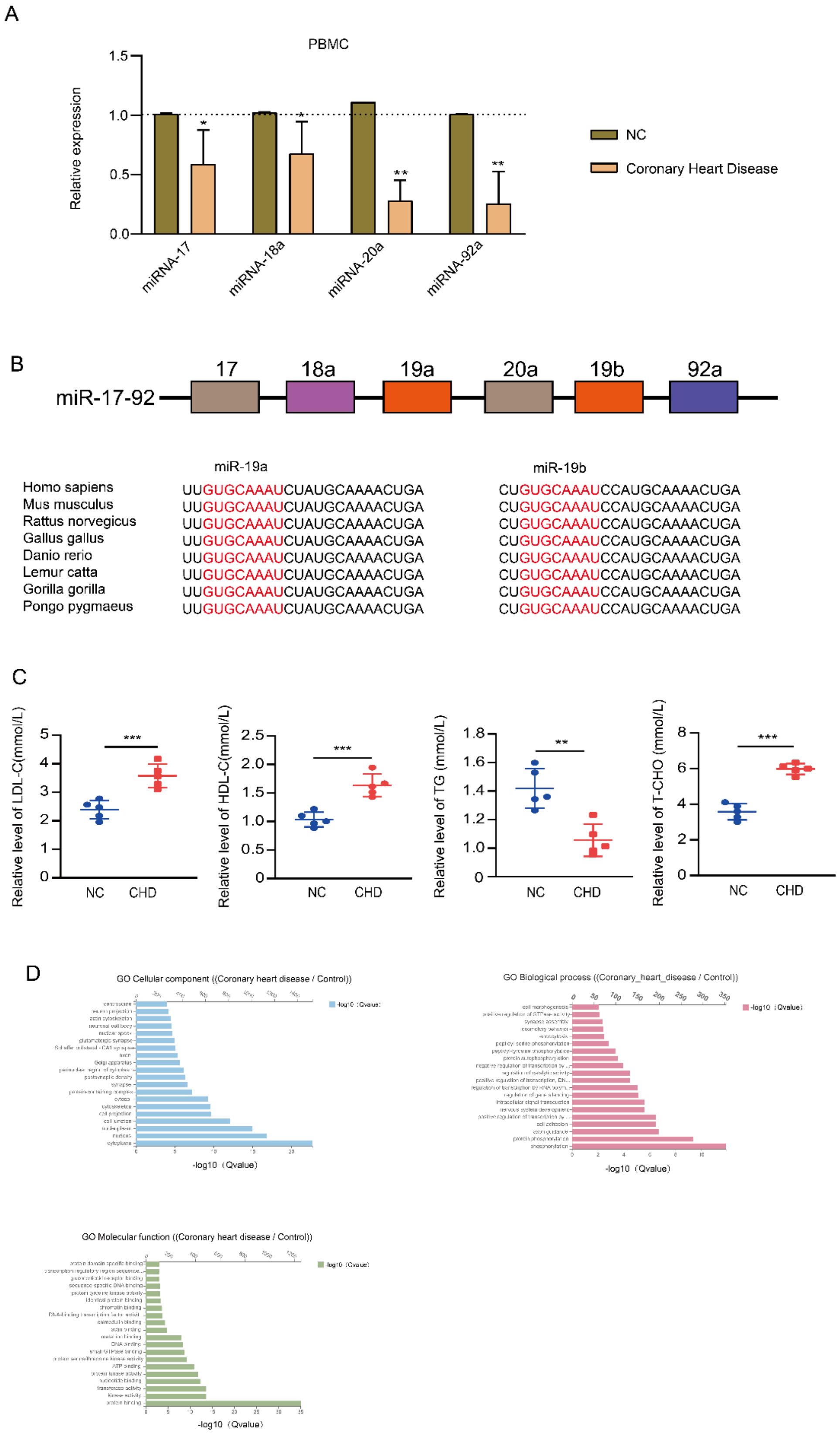
A
PBMC
Relative expression
1.5
1.0
0.5
0.0
miRNA-17
miRNA-18a
miRNA-20a
miRNA-92a
NC
Coronary Heart Disease
B
miR-17-92
17
18a
19a
20a
19b
92a
miR-19a
miR-19b
Homo sapiens UUGUGCAAAUCUAUGCAAAACUGA CUGUGCAAAUCCAUGCAAAACUGA
Mus musculus UUGUGCAAAUCUAUGCAAAACUGA CUGUGCAAAUCCAUGCAAAACUGA
Rattus norvegicus UUGUGCAAAUCUAUGCAAAACUGA CUGUGCAAAUCCAUGCAAAACUGA
Gallus gallus UUGUGCAAAUCUAUGCAAAACUGA CUGUGCAAAUCCAUGCAAAACUGA
Danio rerio UUGUGCAAAUCUAUGCAAAACUGA CUGUGCAAAUCCAUGCAAAACUGA
Lemur catta UUGUGCAAAUCUAUGCAAAACUGA CUGUGCAAAUCCAUGCAAAACUGA
Gorilla gorilla UUGUGCAAAUCUAUGCAAAACUGA CUGUGCAAAUCCAUGCAAAACUGA
Pongo pygmaeus UUGUGCAAAUCUAUGCAAAACUGA CUGUGCAAAUCCAUGCAAAACUGA
C
Relative level of LDL-C(mmol/L)
Relative level of HDL-C(mmol/L)
Relative level of TG (mmol/L)
Relative level of T-CHO (mmol/L)
NC
CHD
D
GO Cellular component ((Coronary heart disease / Control))
GO Biological process ((Coronary_heart_disease / Control))
GO Molecular function ((Coronary heart disease / Control))
-log10（Qvalue）

Figure S2. A. Expression of miR-17, miR-18a, miR-20a and miR-92a detected by qRT-PCR in PBMC of CHD patients and healthy people. B. Biochemical indicators of CHD patients and healthy people. C. Statistics of GO entries enriched with differential microRNAs were enriched between CHD mice and normal mice. Note: BP: BP enrichment, cellular component (CC): CC enrichment, and molecular function (MF): MF enrichment.

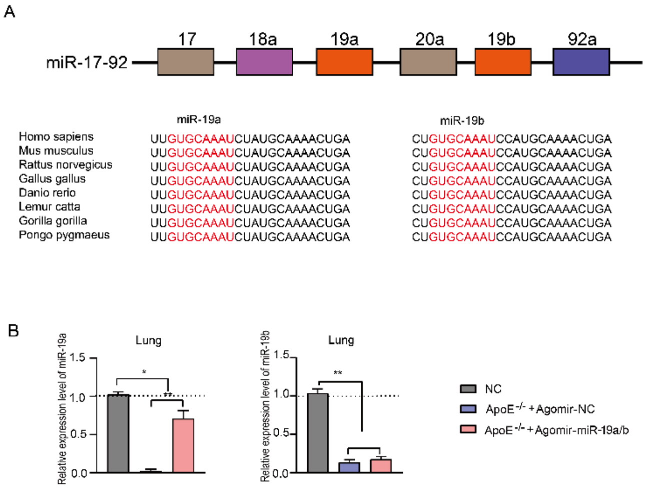

Figure S3. A. Gene structure of the miR-17–92 cluster and conserved sequences of miR-19a and miR-19b across species. Seed sequences are highlighted. B. Expression of miR-19a detected by qRT-PCR in lungs of NC mice, ApoE-/- + agomir-NC mice, and ApoE-/- + agomir-miR-19a/b-treated mice.

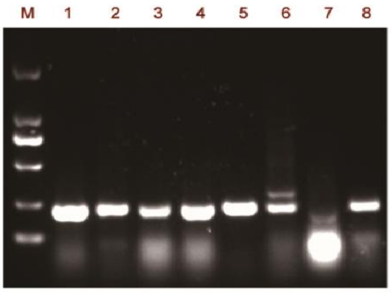

Figure S4. M represents DL2000 marker; lanes 1to 6 represents lung, heart, liver, spleen, kidney, brain, lane 7: Negative control; lane 8: Positive control;

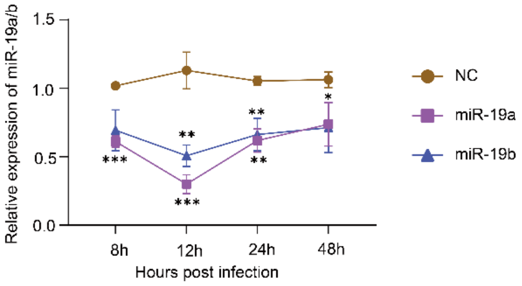

Figure S5. the expression of miR-19a/b was time- and dose-dependent on NDV virus infection detected by quantitative analysis. Asterisks denote the significance levels: * p<0.05; ** p<0.01; *** p<0.001.

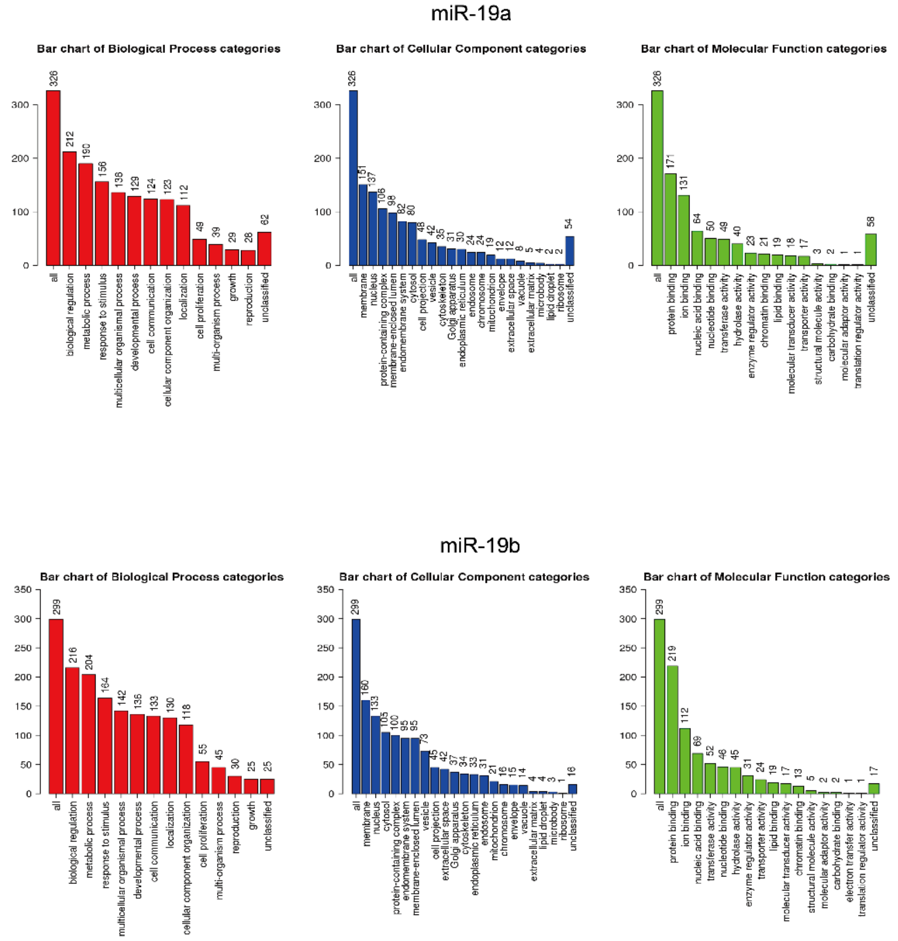

Figure S6. Statistics of GO entries enriched with miR-19a and miR-19b target genes.

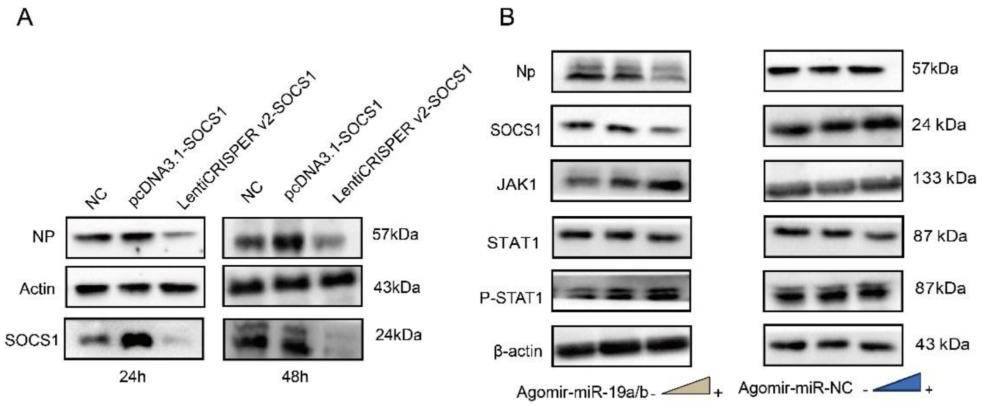

Figure S7.A. A549 cells were transfected with pcDNA3.1-SOCS1, lenti-crisprv2-SOCS1 and empty vector for 48 hours, and then infected with BJ05/H1N1 24h.p.i. western blot detection of NP level in A549 cells were transfected with pcDNA3.1-SOCS1, lenti-crisprv2-SOCS1 and empty vector in different time. B. A549 cells were transfected with agomir-miR-19a/b and agomir-NC at different concentrations and after infected with BJ05/H1N1 24h.p.i, then detected NP, M1, SOCS1, JAK2, STAT1 and p-STAT1 expression levels were analyzed using Western blot.

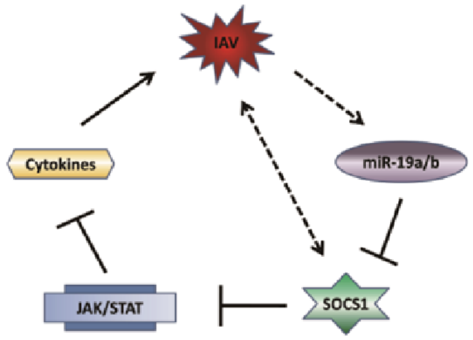

Figure S8. C. A hypothetical model for the role of miR-19/SOCS1 axis in IAV induced inflammatory cytokine expression. Solid arrows, signaling pathways identified in this study; broken arrows, potential signaling pathways.

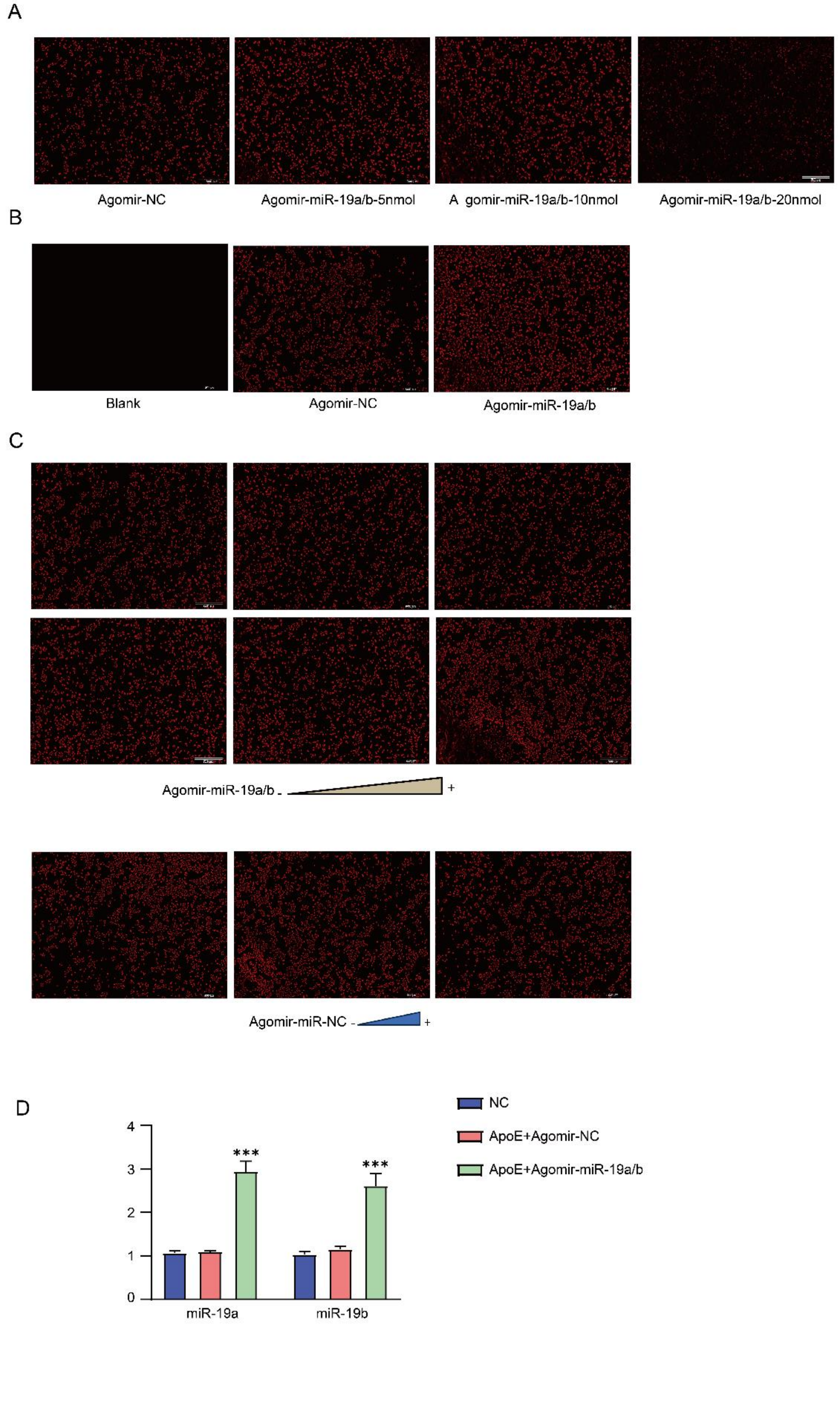

Figure S9. A. Transfection efficiency of Fig.4A. B. Transfection efficiency of Fig.4B. C. Transfection efficiency of Fig. 6C. D. Transfection efficiency of Fig.5D.

Targets	Primer sequence
β-actin F	CACCAGTTCGCCATGGAT
β-actin R	CCTCGTCACCCACATAGGAG
SOCS1 F	TTGGAGGGAGCGGATGGGTGTAG
SOCS1 R	AGAGGTAGGAGGTGCGAGTTCAGGTC
IFN-α F	GAAGGACAGGAAGGATTTGGA
IFN-α R	TGAGCCTTCTGGBATCTGTTGGT
TNF-α F	CATCTTCTCAAAATTCGAGTGACAA
TNF-α R	TGGGAGTAGACAAGGTACAACCC
IL-β F	CAACCAACAAGTGATATTCTCCATG
IL-β R	GATCCACACTCTCCAGCTGCA
IL-6 F	GAGGATACCACTCCCAACAGACC
IL-6 R	AAGTGCATCATCGTTGTTCATACA
U6 F	CTCGCTTCGGCAGCACATATACT
U6 R	CGCTTCACGAATTTGCGTGT
GAPDH F	RCTCAGTGTAGCCCAGGATGC
GAPDH R	FACCACCATGGAGAAGGCTGG
SOCS1WT F	GGATCTTCCAGAGATGAGCTCTGTGGG TACCCTCCCCGG
SOCS1WT R	CTGCCGTTCGACGATCTCGAGAAAAAA AAAACTTTCATAATAAAGTTTATTACC
SOCS1-MUT F	TCAAACGTGTAACCAGGGGTTGGGGG AGGGTCT
SOCS1-MUT R	CCCTGGTTACACGTTTGATACTGGGTAT ATGTAAACATGAAGAGG

Figure S10. Primer sequence
