# Supplementary material for: Therapeutic role of miR-19a/b protection from influenza virus infection in patients with coronary heart disease
Source: Mol Ther Nucleic Acids. 2024 Feb 15;35(1):102149. doi: 10.1016/j.omtn.2024.102149 (PMC10907223; doi:10.1016/j.omtn.2024.102149)
Supplement: Document S2. Article plus supplemental information [file mmc2.pdf]

Molecular Therapy
Nucleic Acids
Original Article

# Therapeutic role of miR-19a/b protection from influenza virus infection in patients with coronary heart disease

Yanan Xing,[1,2,6] Lin Chen,[3,6] Bin Hu,[1,2] Yi Li,[1,2] Huan Mai,[4] Gaojian Li,[1,2] Shuyi Han,[1,2] Ye Wang,[1,2] Yanyi Huang,[1,2] Ying Tian,[5] Wei Zhang,[5] Yan Gao,[4] and Hongxuan He[1]

[1]Institute of Zoology, Chinese Academy of Sciences, Beijing, China; [2]University of Chinese Academy of Sciences, Beijing, China; [3]CAS Center for Excellence in Nanoscience, National Center for Nanoscience and Technology, Beijing, China; [4]Department of Infectious Diseases, Peking University People's Hospital, Beijing, China; [5]Beijing Wildlife Rescue and Rehabilitation Center, Beijing, China

**Patients with pre-existing medical conditions are at a heightened risk of contracting severe acute respiratory syndrome (SARS), SARS-CoV-2, and influenza viruses, which can result in more severe disease progression and increased mortality rates. Nevertheless, the molecular mechanism behind this phenomenon remained largely unidentified. Here, we found that microRNA-19a/b (miR-19a/b), which is a constituent of the miR-17-92 cluster, exhibits reduced expression levels in patients with coronary heart disease in comparison to healthy individuals. The downregulation of miR-19a/b has been observed to facilitate the replication of influenza A virus (IAV). miR-19a/b can effectively inhibit IAV replication by targeting and reducing the expression of SOCS1, as observed in cell-based and coronary heart disease mouse models. This mechanism leads to the alleviation of the inhibitory effect of SOCS1 on the interferon (IFN)/JAK/STAT signaling pathway. The results indicate that the IAV employs a unique approach to inhibit the host's type I IFN-mediated antiviral immune responses by decreasing miR-19a/b. These findings provide additional insights into the underlying mechanisms of susceptibility to flu in patients with coronary heart disease. miR-19a/b can be considered as a preventative/therapy strategy for patients with coronary heart disease against influenza virus infection.**

## INTRODUCTION

The COVID-19 pandemic has resulted in unparalleled worldwide disruption. The seasonal cycle of various infectious diseases, including influenza, has been disrupted globally since early 2020 due to changes in exposure patterns and mobility (Figure S1). Influenza A virus (IAV) is a major and annual epidemic respiratory pathogen associated with serious economic and health issues.[1] It belongs to the *Orthomyxoviridae* family of negative-strand RNA viruses, which is mainly divided into types A, B, C, and D. The high pathogenicity and variability of IAV can significantly increase its potential to cause severe respiratory infections as well as widespread epidemics or influenza,[2,3] which seriously endanger human well-being. The 1918 flu (H1N1) killed nearly 50 million people,[4] and the 2009 H1N1 pandemic killed nearly 17,000 people.[5,6] Though H1N1 pdm09 has been included in the influenza vaccine since 2010, it still propagates in the community annually and was the predominant IAV strain during the 2019–2020 influenza epidemic.[7] Influenza infection is associated with increased mortality in patients with underlying conditions such as cardiovascular disease, chronic kidney disease, diabetes, chronic respiratory disease, and a range of other chronic conditions.[8] The causative mechanism behind adverse outcomes of underlying conditions in patients with influenza was largely unknown.

Coronary heart disease (CHD) is a common disease in middle-aged and elderly people, and the age of its outbreak tends to be younger.[9] Studies have confirmed that high blood pressure, high blood glucose, high blood lipid, smoking, drinking, and genetic factors are intensively related to the risk factors of CHD.[10] Patients with cardiovascular disease have reduced baseline cardiopulmonary and renal function, increasing the risk of respiratory disease. Madjid et al. have demonstrated a clear association between influenza epidemics and the increased rate of deaths from CHD.[11] Several mechanisms have been revealed that show that influenza can increase the risk of cardiovascular events, such as the activation of coagulation cascade, pro-inflammatory mediators, and sympathetic stimulation.[12] However, the specific molecular mechanism through which CHD increased susceptibility to influenza virus remains largely unknown.

MicroRNAs (miRNAs or miRs) are short, single-stranded, non-coding RNAs composed of 21–23 nt that regulate the expression of target genes at the post-transcriptional level by impeding the translation or promoting the degradation of target mRNAs. Expression of virus-derived miRNAs or modulation of host-derived miRNAs has been demonstrated as an effective strategy for viral immune evasion.[13,14]

Received 19 July 2023; accepted 12 February 2024;
https://doi.org/10.1016/j.omtn.2024.102149.

[6]These authors contributed equally

**Correspondence:** Hongxuan He, Institute of Zoology, Chinese Academy of Sciences, Beijing, China.
**E-mail:** hehx@ioz.ac.cn


This is an open access article under the CC BY license (http://creativecommons.org/licenses/by/4.0/).

A

| Coronary heart patient basic information (n=15) | | | Healthy people basic information (n=8) | | |
|---|---|---|---|---|---|
| Climicopathological feature | Number | % of patients | Climicopathological feature | Number | % of patients |
| Age | | | Age | | |
| < 50 | 2 | 13.3% | < 50 | 2 | 25% |
| ≥ 50 | 13 | 86.7% | ≥ 50 | 6 | 75% |
| Gender | | | Gender | | |
| Female | 3 | 20% | Female | 2 | 25% |
| Male | 12 | 80% | Male | 6 | 75% |

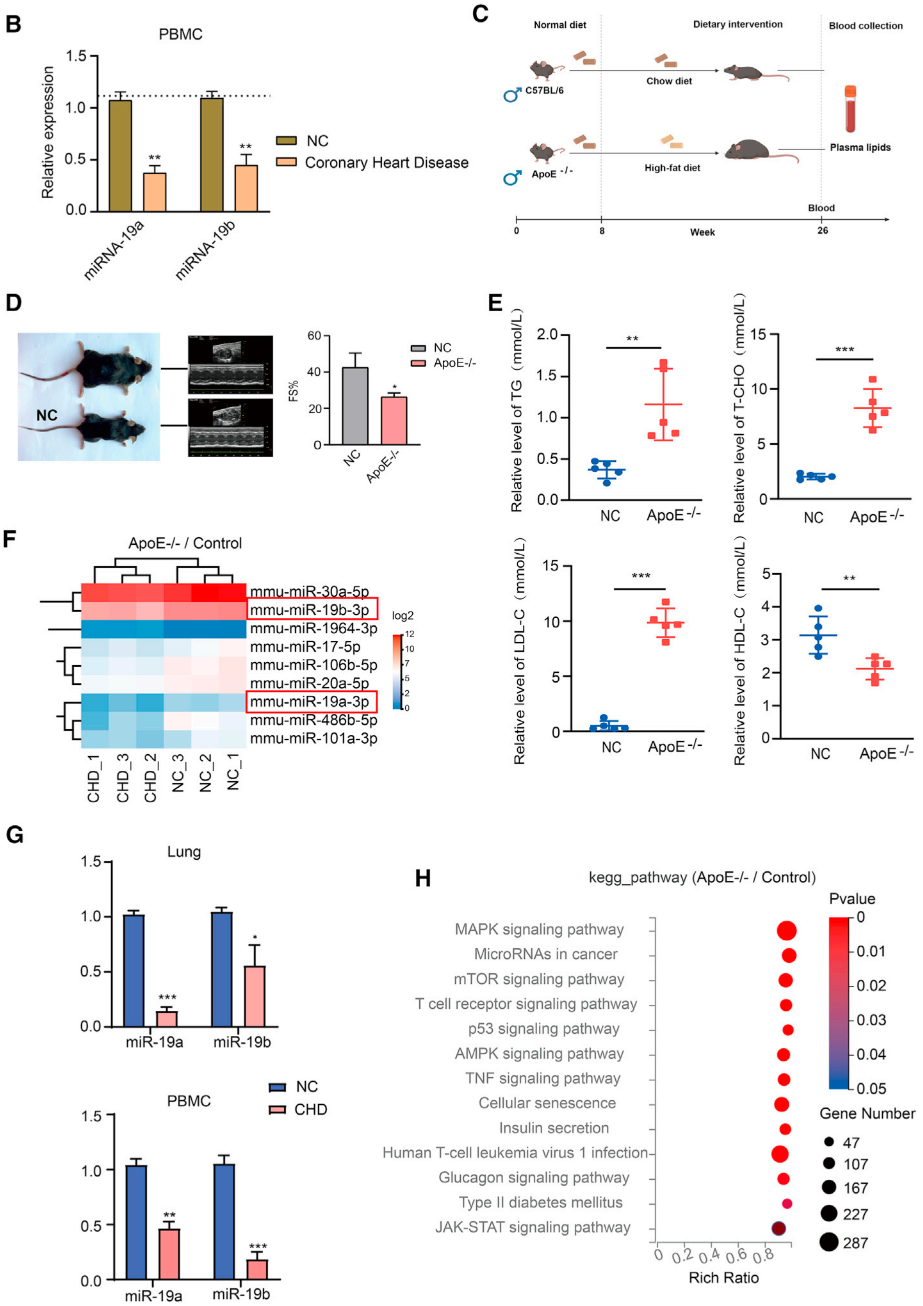


*(legend on next page)*

Conversely, host-derived miRNAs are responsible for the antiviral responses by the direct regulation of virus-derived nucleotides (vcRNA, vmRNA, or vDNA) stability or the modulation of innate and adaptive immunity.[14] Growing evidence has shown that miRNAs are involved in regulating development, apoptosis, host immunity, and viral infection.[15–17] For example, miR-34a can promote influenza virus-mediated apoptosis by binding to BAX16, while miR-let-7c can target and inhibit M1 expression of H1N1 influenza virus.[18] Tambyah et al. observed significant expression changes in 193 miRNAs among 50 patients with severe H1N1 or H3N2 influenza infection.[19] We hypothesized that miRNA expression differences in patients with CHD may exacerbate complications following influenza infection.

The miR-17-92 cluster is polycistronic and situated in the 13q31 region of the human chromosome. Its initial discovery as an oncogene was attributed to its ability to induce transformation.[20] A single transcript transcribes six distinct miRNAs, namely miR-17, miR-18a, miR-19a, miR-19b, miR-20a, and miR-92a. It should be emphasized that despite miR-17-92 being transcribed as a singular transcript, there is a variance in the expression of individual miRNAs due to post-transcriptional processing.[21] Studies indicate that the miR-17-92 gene cluster may play a role in the onset and progression of cardiovascular diseases. Mayer et al.[22] reported that reduced miR-19a expression in the bloodstream is linked to higher mortality rates in individuals with stable coronary artery disease (SCAD). The diminished expression of miR-19a in patients with SCAD may increase the likelihood of mortality due to cardiovascular disease. Singh and colleagues[23] discovered a correlation between reduced plasma miR-19b expression levels in individuals with acute coronary syndrome and an increased likelihood of aspirin resistance and major cardiovascular and cerebrovascular adverse events, including myocardial infarction and stroke. The limited investigation of miR-19 in influenza prompts us to hypothesize that its low expression in individuals with CHD may contribute to the development of severe influenza following infection.

The study revealed a decrease in the expression of miR-19a/b among patients diagnosed with CHD. Further analysis of *in vivo* and *in vitro* infection with IAV revealed that overexpression of miR-19a/b impeded SOCS1, consequently suppressing the release of inflammatory mediators. This observation may offer novel insights into the heightened susceptibility of patients with CHD to influenza.

## RESULTS

### Patients or mice with CHD exhibit low expression of miR-19a and miR-19b

Our study aimed to evaluate the predictive potential of circulating miRNAs in patients with CHD. We recruited 15 patients with CHD and 8 healthy people who had not received antitumor drugs, radiotherapy, immunosuppressants, hypoglycemic drugs, lipid-lowering drugs, vitamin C, aspirin, or antioxidant drugs prior to blood collection, and the work performed was accepted by the research ethics committee. Figure 1A displays the data of the patients with CHD and of the healthy people. Peripheral blood mononuclear cells (PBMCs) were isolated using Ficoll lymphocyte separation medium, and total RNA was extracted from collected blood samples. RT-qPCR was used to quantify miRNA expression in patients with CHD (Figure S2A). The results indicated lower expression of six members of miR17-92 in these patients compared to healthy individuals, with miR-19a and miR-19b showing the most significant decreases (Figure 1B). To assess the consistency of miR-19a and miR-19b in animals, we created a mice model with CHD. $ApoE^{-/-}$ mice were subjected to an 18-week high-fat diet (Figure 1C), resulting in increased body size and weight compared to normal mice. Cardiac contractile ability was assessed using B-ultrasound, while lipid changes were measured using a test kit (Figures 1D and 1E). The results were similar to those of patients with CHD (Figure S2B). miRNA expression profiles were obtained from the lungs of $ApoE^{-/-}$ mice using transcriptome sequencing and bioinformatics techniques, resulting in the identification of 9 differentially expressed miRNAs (Figure 1F). The expression difference of miR-19a and miR-19b was observed in both PBMCs and lung tissues of $ApoE^{-/-}$ mice and normal mice, as depicted in Figure 1G. Axon guidance and focal adhesion were the most enriched signaling pathways, as indicated by the KEGG pathway analysis (Figure 1H). The Gene Ontology (GO) enrichment analysis indicated that intracellular components and membrane-bound organelles were the most pertinent target genes of differentially expressed miRNAs in cell components. Molecular functions primarily encompass protein binding, catalytic activity, and other related functions. At the biological level, the focus is primarily on cellular processes, biological regulation, and metabolic processes (Figure S2C).

### The miR-19a/b complex exhibits *in vivo* attenuation of IAV replication

Our hypothesis posits that miR-19a and miR-19b may play a role in the regulation of influenza virus. To confirm this conjecture, we sought to artificially increase the expression of miR-19a and miR-19b in $ApoE^{-/-}$ mice. miR-19a and miR-19b exhibit a high degree of functional similarity, as they share the same seed sequence (Figure S3A), differing by only one base,[24] so we conducted an experiment to investigate both miRNAs together. This study investigated the effect of an intravenous agomir-miR-19a/b mixture on susceptibility to influenza in $ApoE^{-/-}$ mice; the results are presented in a flow diagram (Figure 2A) and show that the expression levels of miR-19a/b in the PBMCs and lungs of $ApoE^{-/-}$+agomir-NC-treated mice were lower

**Figure 1. Expression of miR-19a/miR-19b in the coronary heart disease of patients and mice**

(A) The basic information of patients with coronary heart disease and healthy people. (B) RT-qPCR of miR-19a and miR-19b in patients with coronary heart disease in PBMCs. n = 15. (C) Schematic diagram of experimental protocol for coronary heart disease mouse model. (D) Myocardial contractility (FS, fractional shortening). (E) Biochemical indicators of NC mice and $ApoE^{-/-}$ mice. (F) $ApoE^{-/-}$ mice and healthy mice of miRNA sequencing in lungs. (G) Scheme of miRNA quantification process. (H) KEGG enrichment of miRNA target genes in lungs of $ApoE^{-/-}$ mice and NC mice. Data are presented as mean ± SD. Asterisks denote the significance levels: *$p < 0.05$, **$p < 0.01$, and ***$p < 0.001$.

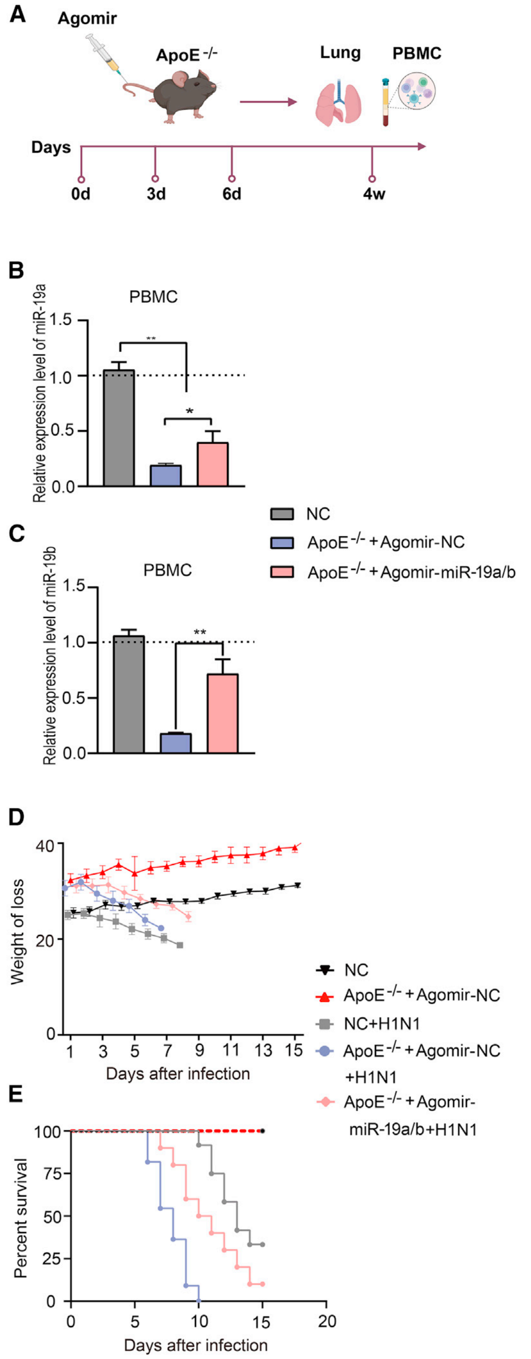


**Figure 2. Direct injection of agomir-miR-19a/b can reduce the mortality of mice infected with influenza**

(A) *ApoE*$^{-/-}$ mice were injected with the mixture of micr*ON* agomir-miR-19a/b and agomir-NC by tail vein once every 3 days, 5 nmol each time, for 3 weeks. (B and C) Expression of miR-19a detected by RT-qPCR in PBMCs of NC mice, *ApoE*$^{-/-}$+ agomir-NC-treated mice, and *ApoE*$^{-/-}$+agomir-miR-19a/b-treated mice. (D) Changes in the weight of five groups (n = 10). (E) Survival curves of five groups (n = 10). Data are presented as mean ± SD. Asterisks denote the significance levels: *p < 0.05, **p < 0.01, and ***p < 0.001.

than in NC mice. However, *ApoE*$^{-/-}$+agomir-miR-19a/b-treated mice group showed higher expression levels of miR-19a/b than the *ApoE*$^{-/-}$+agomir-NC-treated mice group, with significant differences ($p < 0.05$) (Figures 2B, 2C, and S3B). Subsequently, an investigation was conducted to determine the potential impact of miR-19a/b on the mortality and morbidity of IAVs in *vivo*. The wild animal disease research group at the Institute of Zoology, Chinese Academy of Sciences, isolated and preserved the BJ05/H1N1 subtype influenza virus during their previous monitoring efforts. The NC mice, *ApoE*$^{-/-}$+agomir-NC-treated mice, and *ApoE*$^{-/-}$+agomir-miR-19a/b-treated mice were intranasally inoculated with BJ05/H1N1 influenza virus at a dosage of $10^4$ plaque-forming units. According to the findings, the administration of agomir-miR-19a/b to *ApoE*$^{-/-}$ mice effectively suppressed weight loss in the presence of BJ05/H1N1 influenza virus infection. On the contrary, subjects who did not receive agomir-miR-19a/b for *ApoE*$^{-/-}$ mice exhibited elevated levels of weight loss and more severe clinical symptoms, including curling, lethargy, dyspnea, and anorexia. Despite the eventual mortality of all mice inoculated with BJ05/H1N1, the administration of agomir-miR-19a/b injection resulted in a reduction of mortality in *ApoE*$^{-/-}$ mice and an increase in their survival time, as depicted in Figures 2D and 2E. Collectively, these findings suggest that miR-19a/b may ameliorate influenza virus infection symptoms in *ApoE*$^{-/-}$ mice.

## Pathological data pertaining to mice infected with H1N1

In order to gain insight into the pathological characteristics of BJ05/H1N1-infected groups, mice were euthanized at specific time points, including days 1, 2, 3, and 5 post-infection. The observed pathological changes in the NC mice, *ApoE*$^{-/-}$+agomir-NC-treated mice, and *ApoE*$^{-/-}$+agomir-miR-19a/b-treated mice primarily manifested in the lung tissues, characterized by pulmonary hemorrhage and pulmonary edema. The brain, intestine, spleen, and liver did not exhibit apparent lesions. However, the presence of infectious virus particles and viral RNA (vcRNA or vmRNA) was observed (Figure S4). The lung tissue's pathological changes in mice were observed to have significantly worsened over the course of the infection. The pulmonary hemorrhage area was observed to have expanded, and the level of pulmonary edema was found to have escalated. The pulmonary pathological alterations in three cohorts of mice were most severe at the 7-day mark following infection (Figure 3A). The *ApoE*$^{-/-}$+agomir-NC-treated mice group exhibited more pronounced pathological changes compared to those in the *ApoE*$^{-/-}$+agomir-miR-19a/b-treated group (Figure 3B). The *ApoE*$^{-/-}$ +agomir-NC-treated mice group exhibited pulmonary interstitial hyperplasia, alveolar structure disappearance, inflammatory cell infiltration, and significant pathological alterations in the lungs. The *ApoE*$^{-/-}$+agomir-miR-19a/b-treated mice developed lung parenchyma lesions after 5 days of infection, whereas *ApoE*$^{-/-}$+agomir-NC-treated mice showed an expansion of lesion area that persisted until 7 days post-infection (Figure 3B). We further examined the distribution of viral nucleoprotein in lungs. Results showed that virus particles were widely distributed in lung tissues of *ApoE*$^{-/-}$+agomir-NC-treated mice, while the distribution of virus particles was limited in the *ApoE*$^{-/-}$+agomir-miR-19a/b-treated group (Figures 3C and 3D). The distribution of viral nucleoprotein

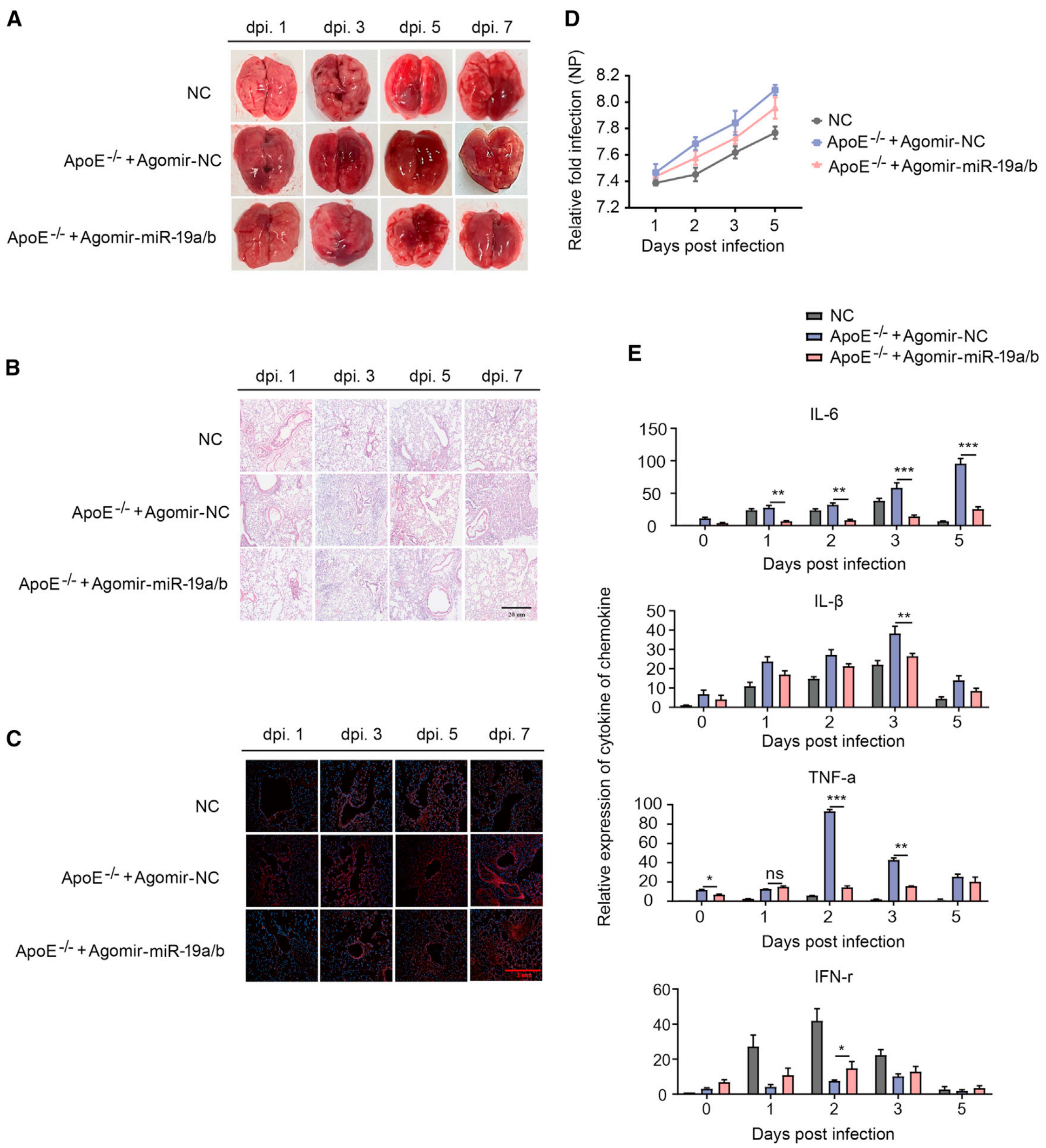


**Figure 3. Pathogenicity of lungs in BJ05/H1N1 influenza virus-infected mice**

(A–C) From day 1 to 5 after influenza virus infection in NC mice, *ApoE*$^{-/-}$+agomir-NC-treated mice, and *ApoE*$^{-/-}$+agomir-miR-19a/b-treated mice, pathological lesions in the lungs of mice, H&E staining, and immunofluorescence. (D) Quantification of immunofluorescence of NP protein. (E) The expression of inflammatory factors in NC mice, *ApoE*$^{-/-}$+agomir-NC-treated mice, and *ApoE*$^{-/-}$+agomir-miR-19a/b-treated mice was quantitatively analyzed. Asterisks denote the significance levels: *$p < 0.05$, **$p < 0.01$, and ***$p < 0.001$.

in lungs was further analyzed. The study found that virus particles were prevalent in the lung tissues of *ApoE*$^{-/-}$+agomir-NC-treated mice, but were restricted in *ApoE*$^{-/-}$+agomir-miR-19a/b-treated mice (Figures 3C and 3D). Mice injected with agomir-miR-19a/b exhibited greater resistance to BJ05/H1N1 infection compared to mice with CHD. This suggests that miR-19a/b may impede the replication of influenza virus. In general, the resistance of *ApoE*$^{-/-}$ mice injected with agomir-miR-19a/b to BJ05/H1N1 infection was stronger than that of *ApoE*$^{-/-}$+agomir-NC-treated mice, indicating that miR-19a/b may hamper the replication of influenza virus. Moreover, the extent of the lesions increased with the duration of infection.

The present study delved deeper into the expression of interferon γ (IFN-γ), tumor necrosis factor α (TNF-α), interleukin-1β (IL-1β),

and IL-6, which are known to exert a crucial inhibitory effect on virus replication. The study findings indicate that the mice treated agomir-miR-19a/b exhibited elevated levels of IFN-$\gamma$ compared to the mice with CHD, as illustrated in Figure 3E. Furthermore, the significant pro-inflammatory factors IL-1$\beta$ and IL-6 exhibited an increase in mice with CHD. An increase in TNF-$\alpha$ expression was noted in both $ApoE^{-/-}$+agomir-NC-treated mice and $ApoE^{-/-}$+agomir-miR-19a/b-treated mice. However, the rate of TNF-$\alpha$ upregulation in $ApoE^{-/-}$+agomir-miR-19a/b-treated mice was comparatively slower than in CHD mice. The findings indicate that $ApoE^{-/-}$ mice treated with agomir-miR-19a/b may elicit antiviral responses more effectively than $ApoE^{-/-}$+agomir-NC-treated mice.

#### miR-19a/b suppresses influenza virus replication

Following the successful demonstration of the ability of miR-19a/b to decrease mortality and morbidity in $ApoE^{-/-}$ mice infected with influenza in *vivo*, our subsequent inquiry focused on the potential impact of miR-19a/b on the inhibition of IAV replication *in vitro*. In order to achieve this objective, A549 cells were transfected with varying concentrations of agomir-miR19-a/b and subsequently exposed to BJ05/H1N1 infection at multiplicity of infection (MOI) = 1. The replication of the virus was monitored at 24 h and 48 h post-infection (hpi). The findings indicate that miR-19a/b, at varying concentrations, effectively inhibited BJ05/H1N1 replication at both 24 and 48 hpi. Notably, the most potent inhibition of BJ05/H1N1 replication was observed with the use of 20 nM agomir-miR-19a/b, as depicted in Figure 4A. Transfection efficiency is shown in Figure S9A. In order to investigate the influence of miR-19a/b on influenza virus infection, we conducted an assessment of virus infection utilizing agomir-miR-19a/b. According to the data, the agomir-miR-19a/b exhibited a significant inhibitory effect on the expression of viral NP and M1 (Figure 4B). Transfection efficiency is shown in Figure S9B. Subsequent research results showed that miR19a/b has the ability to inhibit the replication of BJ05/H1N1 in A549 cells.

Several studies have examined miRNA expression following infection with IAV. The expression of miR-30 family members, specifically miR-30, has been observed to be regulated by IAV attack.[25] Subsequently, we conducted an assessment to determine if IAV infection can regulate miR-19a/b in relevant cellular models. To this end, we assessed the expression levels of miR-19a/b in A549 cells at various time intervals following BJ05/H1N1 infection. A notable observation was made regarding viral infection, particularly in the initial phase of infection. The expression of miR-19a/b was observed to be significantly reduced in A549 cells that were infected with BJ05/H1N1. As the innate antiviral immune response takes place during the early stages of IAV infection, it is hypothesized that miR-19a/b may modulate influenza infection by regulating the innate antiviral immune response. The levels of miR-19a/b were measured in A549 cells that were infected with varying MOIs of BJ05/H1N1. The results indicate that the expression of miR-19a/b decreased in a dose-dependent manner in A549 cells infected with BJ05/H1N1, as shown in Figures 4C and 4D. We used another virus, NDV1/Pigeon/BJ/2023, to infect DF1 cells 24 hpi and obtained similar results in Figure S5. Collectively, these findings indicate that miR-19a and miR-19b exerted inhibitory effects on influenza virus replication at the cellular level.

#### The suppressor of cytokine signaling proteins 1 has been identified as a target of miR-19a/b

To investigate the mechanism by which miR-19a/b inhibits influenza replication, we identified their potential targets. RNA hybrid sequence analyses suggest that the miR-17-92 cluster does not directly target IAV viral genomes. As a result, we propose that miR-17-92 may regulate IAV replication by modulating the cellular signaling pathway. The TargetScan 7.2 and miRBase databases were utilized for identifying potential targets in bioinformatics analysis (Figure 5A). SOCS1, which has potential binding sites, is a significant target involved in the antiviral response (Figure 5B). miR-19a/b was identified as a potential target in the 3′ UTR region of SOCS1 mRNA through comparison analysis, and we analyzed the binding site UUUGCACA between miR-19a/b and the SOCS1 mRNA 3′ UTR region through NCBI. Wild-type (WT)-SOCS1 and mutated (MUT)-SOCS1 were designed and evaluated by dual-luciferase reporter experiment. The results showed that the pmirGLO-SOCS1-WT+miR-19a/b group had the lowest firefly/Renilla luciferase expression, whereas the binding site MUT-SOCS1 did not downregulate firefly/Renilla luciferase expression. It was confirmed that miR-19a/b inhibited SOCS1 expression by targeting the SOCS1 mRNA 3′ UTR (Figure 5C). Previous studies have demonstrated that miR-19a/b targets the gene SOCS1.[26] The target genes of miR-19a/b were subjected to GO and pathway enrichment analysis. A total of 300 target genes were analyzed using the WebGestalt online tool, which yielded a set of biological processes associated with these target genes. The biological processes encompass various categories such as endogenous stimulus responses, cell growth regulation, proliferation, and metabolic processes (Figure S6A). We verified the correlation between IAV replication and SOCS1 expression (Figure 5E). The infection of BJ05/H1N1 in the expression of SOCS1 protein in a manner that was dependent on time.

SOCS1 expression was assessed at various time intervals during BJ05/H1N1 infection in A549 cells. SOCS1 expression was found to be upregulated in a time-dependent manner in A549 cells infected with BJ05/H1N1, particularly in the early stages of infection (Figure 5F). The level of SOCS1 was detected in A549 cells infected with BJ05/H1N1 at varying MOIs. The study found that the expression of SOCS1 increased in a dose-dependent manner in A549 cells infected with BJ05/H1N1 (Figure 5G). SOCS1 protein expression was observed in the lungs of three groups of mice. Following agomir-miR-19a/b treatment, $ApoE^{-/-}$ mice exhibited inhibited SOCS1 expression (Figure 5D). Transfection efficiency is shown in Figure S9D.

#### miR-19a/b enhances JAK/STAT signaling pathway activation

We used lung tissues of BJ05/H1N1 virus mice infected for 5 days, including $ApoE^{-/-}$+agomir-NC, $ApoE^{-/-}$+agomir-miR19a/b, and NC, for transcriptome sequencing. By analyzing the three KEGG pathways of $ApoE^{-/-}$+agomir-NC/NC, $ApoE^{-/-}$+agomir-miR19a/b/NC, and $ApoE^{-/-}$+agomir-miR19a/b/$ApoE^{-/-}$+agomir-NC, it

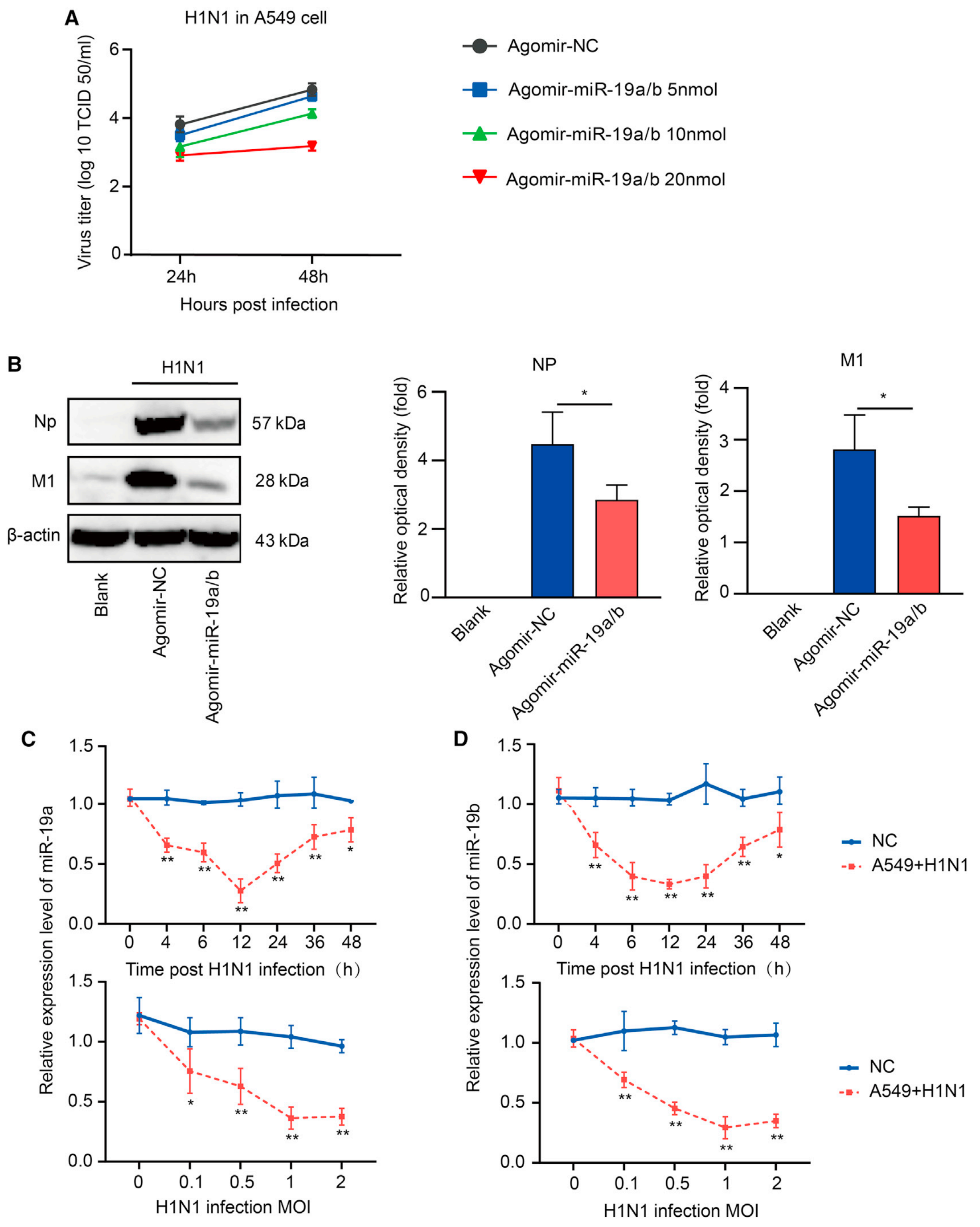


**Figure 4. miR-19a/b inhibits influenza virus replication in A549 cells**

(A) Different concentrations of agomir-miR-19a/b influenced H1N1 by $TCID_{50}$. (B) Expression of NP or M1 after infection with H1N1 in NC, agomir-NC, and agomir-miR-19a/b of cells. (C and D) The expression of miR-19a/b was time- and dose dependent on H1N1 virus infection detected by quantitative analysis. Asterisks denote the significance levels: *p < 0.05, **p < 0.01, and ***p < 0.001.

was found that differential genes were enriched in the JAK/STAT pathway (Figure 6A). It has been demonstrated that NS and M proteins exert a negative regulatory effect on JAK/STAT signal transduction by upregulating the expression of SOCS1 and SOCS3.[27] Therefore, we propose that miR-19a/b may upregulate JAK/STAT signal transduction by suppressing SOCS1 expression, leading to inhibition

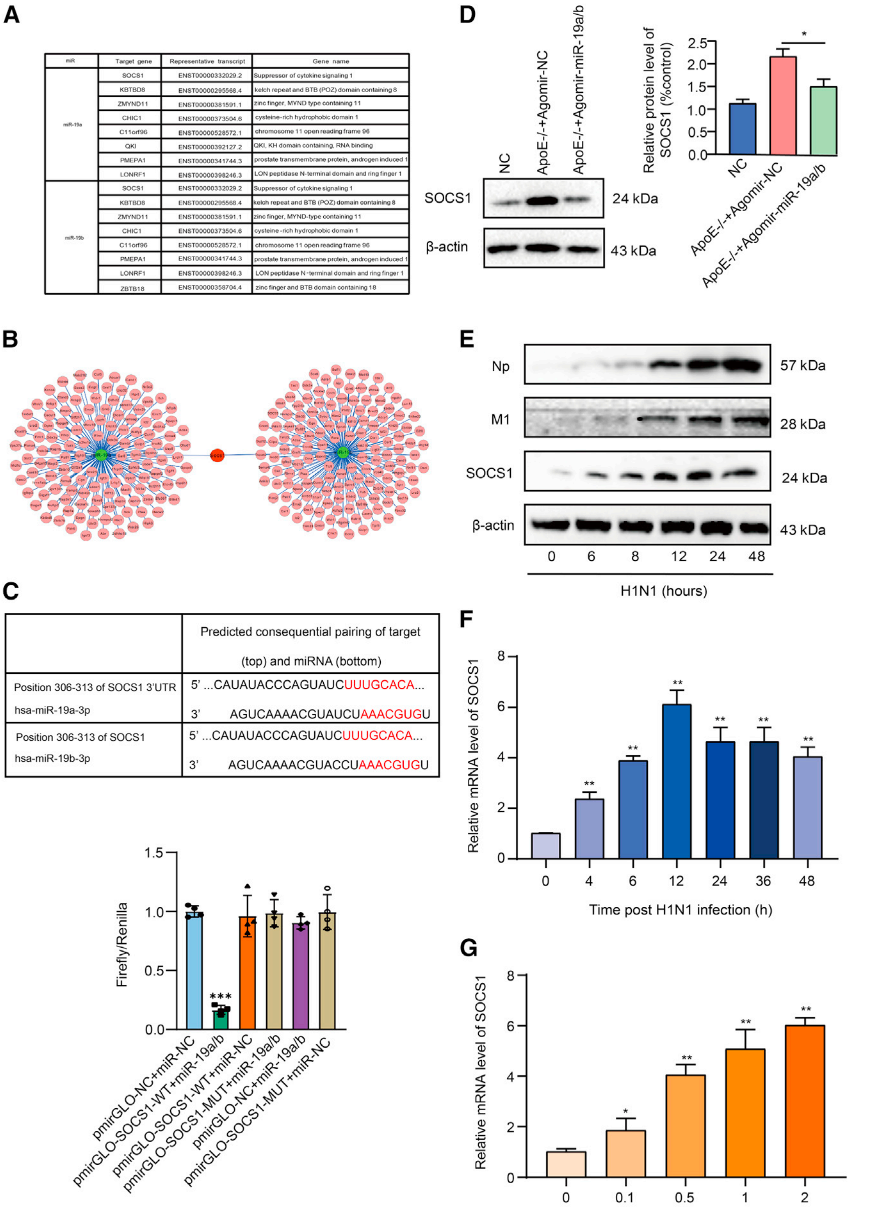


**Figure 5. microRNA-19a/b target site analysis**

(A) Related target genes of miR-19a/b. (B) miR-19a and miR-19b target interaction network. Nodes: genes or miRNAs, lines: connections, green: miR-19a and miR-19b, pink: target genes, and red: SOCS1. (C) The binding region of SOCS1 3′ UTR and miR-19a/b; dual-luciferase reporter experiment. (D) The lung of NC mice, *ApoE*$^{-/-}$+agomir-NC-treated mice, and *ApoE*$^{-/-}$+agomir-miR-19a/b-treated mice that expressed SOCS1 as detected by western blotting. (E) The protein expression levels of NP, M1 and socs1 at different times after influenza virus infection of A549 cells. (F and G) The expression of SOCS1 was time- and dose dependent on influenza infection detected by quantitative analysis. Asterisks denote the significance levels: *p < 0.05, **p < 0.01, and ***p < 0.001.

of viral infection. To examine the impact of miR-19a/b on SOCS1 protein levels, A549 cells were both infected with BJ05/H1N1 (MOI = 1) and transfected with varying concentrations of agomir-miR-19a/b. Transfecting agomir-miR-19a/b into A549 cells resulted in a significant decrease in the expression of SOCS1 and influenza protein. miR-19a/b has demonstrated a dose-dependent suppression of SOCS1 protein, and there was no change in the agomir-NC group (Figure 6B).

The activation of the STAT1 signaling cascade during IAV infection was examined by analyzing the phosphorylation of STAT1 at tyrosine 701. This site is crucial for full activation of STAT1 and is specifically targeted in a manner that is dependent on STAT1. Phosphorylation of STAT1 was observed following infection of A549 cells with BJ05/H1N1 virus at an MOI of 1. Increasing the concentration of agomir-miR-19a/b resulted in a greater inhibition of SOCS and an increase in the expression of JAK and p-STAT1. In order to verify that miR-19a/b mainly inhibits viruses by targeting SOCS1, we first constructed the SOCS1-knockout (KO) cells and, through re-expression of SOCS1$^{WT}$ in SOCS1-KO cells, constructed the exogenous SOCS1 overexpression cell line. The two groups were transfected with SOCS1$^{WT}$+miR-19a/b and SOCS1$^{WT}$+miR-NC, respectively, and then infected with H1N1 virus at MOI = 1 for 24 h, and we detected their protein expression levels to compare the differences. The results showed that the NP levels did not downregulate in exogenous SOCS1 cells, indicating that miR-19a/b mainly targeted SOCS1 to inhibit viral replication.

Inflammatory cytokine expression was assessed at various time intervals, revealing lower levels in the simulant treatment group compared to the CHD group (Figure 6E). The findings suggest that miR-19a/b can activate the JAK/STAT pathway by targeting SOCS1.

### SOCS1 promotes influenza virus replication

To further determine the role of SOCS1 in IAV infection, pcDNA3.1-SOCS1, lenti-crisprv2-SOCS1, and empty vector were transfected into A549 cells and overexpressed, and KO SOCS1 cell lines were constructed, which were infected with BJ05/H1N1. As shown in Figure 7A, overexpression of SOCS1 significantly promoted the replication of BJ05/H1N1 and reached a peak value at 36 h, whereas SOCS1 deletion inhibited the expression of NP (Figure 7B), which was consistent with the results of western blot (Figures 7C and 7D).

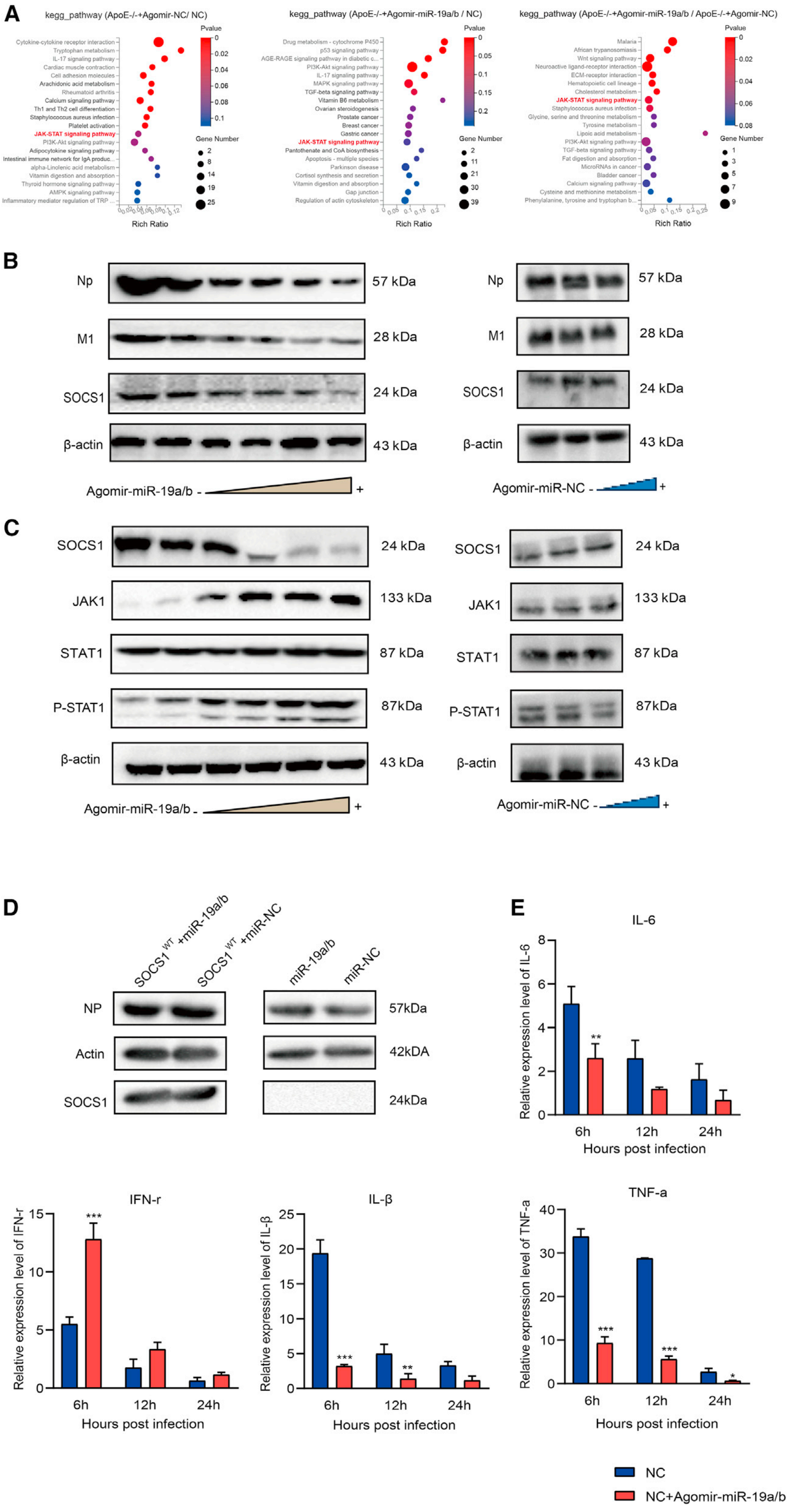


**Figure 6. Overexpression miR-19a/b inhibited the expression of SOCS1**

(A) KEGG pathways of *ApoE*$^{-/-}$+agomir-NC/NC, *ApoE*$^{-/-}$+agomir-miR19a/b/NC, and *ApoE*$^{-/-}$+agomir-miR19a/b/*ApoE*$^{-/-}$+agomir-NC mice. (B) A549 cells were transfected with agomir-miR-19a/b and agomir-NC at different concentrations and 24 hpi with BJ05/H1N1, and then detected NP, M1, and SOCS1 expression levels were analyzed using western blot. (C) Western blot detection of SOCS1, JAK2, STAT1, and p-STAT1 in A549 cells treated as in (B). (D) Western blot detection of NP and SOCS1 in four groups of A549 cells 24 hpi with BJ05/H1N1. (E) Quantitative analysis of cytokines 6, 12, and 24 h after influenza virus infection of A549 cells. Data represent means ± SEM. p values were determined by one-way ANOVA comparisons test. Asterisks denote the significance levels: $^*p < 0.05$, $^{**}p < 0.01$, and $^{***}p < 0.001$.

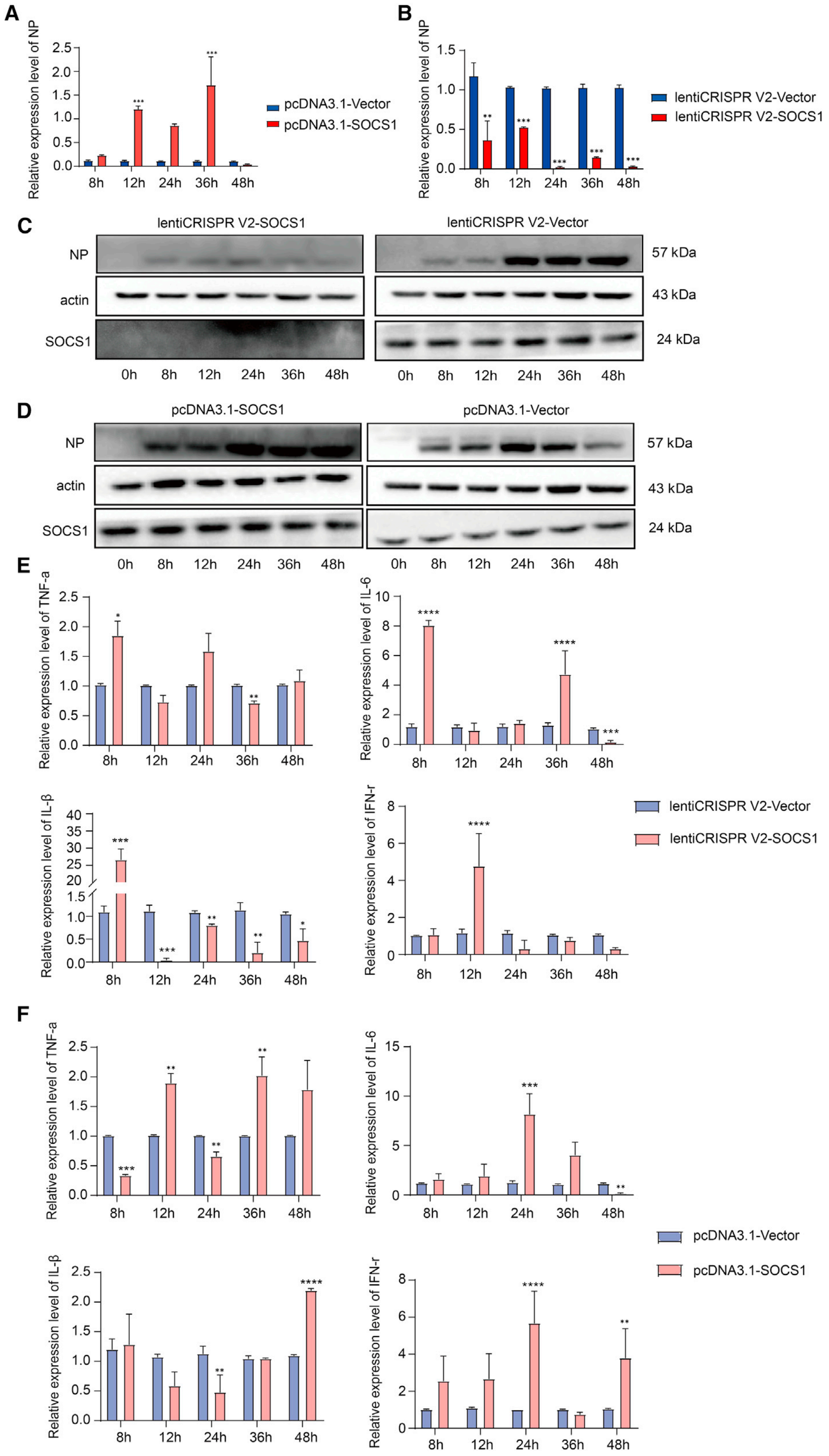


*(legend on next page)*

We use one more influenza virus (A/environment/Qinghai/1/2008/H5N1) strain to verify the major conclusion. A549 cells were transfected with lenti-crisprv2-SOCS1 or pcDNA3.1-SOCS1 for 48 h and then infected with H5N1 virus. At 24 and 48 hpi, cells were washed, and the protein was extracted and detected by western blot (Figure S7). RT-qPCR was used to evaluate the expression of inflammatory cytokines at different time intervals, showing that SOCS1 KO could significantly inhibit the expression of inflammatory cytokines (Figures 7E and 7F). These results clearly identify the role of SOCS1 as a viral factor in the cellular response to BJ05/H1N1 infection.

## DISCUSSION

Individuals with CHD are at heightened risk for IAV infection. miR-19a and miR-19b expression is downregulated in patients with CHD compared to healthy individuals. Studies have demonstrated that reduced expression of miR-19a and miR-19b can result in heightened susceptibility to IAV infection, both *in vivo* and *in vitro*. Our study indicates that reduced levels of miR-19a and miR-19b may exacerbate the incidence and fatality of influenza virus infection. This offers a novel perspective on comprehending the populace's inclination toward influenza.

The miR-17-92 gene cluster is conserved and has been linked to the development of multiple organ dysfunction syndrome and tumorigenesis in mammals. The six members play significant roles in various settings such as normal development, immune disease, cardiovascular disease, neurodegenerative disease, and aging, among others.[28] Recent studies have established the crucial involvement of miRNAs in the innate immune response against viruses. Shimakami et al.[29] found that miR-122 can directly impact the RNA genome of Hepatitis C virus (HCV) during infection, leading to changes in genome stability and increased viral replication. Conversely, certain research groups have yielded contradictory outcomes. Lanford et al.[30] demonstrated that miR-122 can impede the replication of HCV by inducing the degradation of its RNA genome. hsa-miR-122, hsa-miR-199a, hsa-miR-448, hsa-miR-196, and hsa-let-7b have been identified as direct inhibitors of HCV replication. miR-let-7c inhibits the expression of H1N1 influenza virus M1 protein and thereby affects the replication of influenza virus in human lung epithelial cells.[31] To date, limited research has explored the possible involvement of the miR-17-92 cluster in the antiviral mechanism. We investigated the mechanism underlying the inhibitory effect of miR-19a and miR-19b on virus infection. SOCS1 was identified as a target of miR-19a and miR-19b in the present study. The SOCS family plays a significant role in regulating the innate immune response triggered by microbial pathogens. Their main role is to regulate negative feedback of cytokine signal transduction through JAK/STAT and other signal pathways.[18,32] Studies indicate that miRNA expression profiles of the host or virus may alter during viral infections, and certain miRNAs can regulate SOCS protein expression to control innate immune pathways.[33–35] Certain miRNAs, both viral and host derived, can regulate viral replication by targeting either the viral genome or host genes. This has been documented in previous studies.[36,37] During infectious bursal disease (IBD) infection, miR-155 in the host can hinder the expression of SOCS1 and TANK, which ultimately restrains IBDV replication by promoting IFN-I-mediated antiviral response.[38] TGEV infection induces endoplasmic reticulum stress and upregulates IRE1α expression, leading to a decrease in host miR-30a-5p levels. This reduction in miR-30a-5p inhibits antiviral responses by decreasing SOCS1 and SOCS3 expression, thereby promoting TGEV replication.[39]

The precise function of miR19a/b in influenza remains uncertain. This study presents evidence that miR19a/b, through targeting SOCS1, plays a role in mediating influenza-induced cytokine storms both *in vitro* and *in vivo*. Subsequent studies have verified that late-stage influenza virus-infected cells can increase miR19a/b expression and suppress SOCS1 expression, thus stimulating the JAK/STAT signaling pathway and augmenting the antiviral properties of IFN. Overexpression of SOCS1 reduced phosphorylation of JAK1 and STAT1, leading to inhibition of IFN-I-induced antiviral and antiproliferative responses. The expression of miR19a/b mRNA was found to be suppressed during the initial stages of viral replication (as early as 12 h) in individuals infected with influenza. This observation suggests that the virus may have developed mechanisms to evade the immune system in order to facilitate its replication.

Comprehensive clinical research suggests that influenza virus susceptibility in CHD may be attributed to several mechanisms. Firstly, viral infection can act as an inflammatory stimulus, impacting the body's coagulation function and leading to a decline in antithrombin-III function, thereby creating favorable conditions for the formation of coronary atherosclerotic plaques. Secondly, viral infection can affect the formation of blood lipids, resulting in an increase in the level of low-density lipoprotein and the occurrence of atherosclerotic plaques. Lastly, the body's response to stress after virus infection, combined with the virus itself acting as an inflammatory stimulus, can cause the instability and rupture of atherosclerotic plaques, inducing the expression of IL-8, TNF-α, and other factors. The activation of other cytokines results in the promotion of a heightened inflammatory response, which in turn triggers the rupture of atherosclerotic plaques and subsequently leads to an acute CHD episode. The expression level of miR-19a/b is observed to be downregulated in cases of CHD, accompanied by an increase in the expression level of SOCS1. The protein SOCS1 functions as an inhibitor of the

**Figure 7. SOCS1 knockdown inhibited NP expression**

A549 cells were transfected with pcDNA3.1-SOCS1, lenti-crisprv2-SOCS1, and empty vector for 48 h and then infected with BJ05/H1N1 24 hpi. (A and B) The NP level was detected by RT-qPCR at different times. (C and D) Western blot detection of NP level in A549 cells transfected with pcDNA3.1-SOCS1, lenti-crisprv2-SOCS1, and empty vector at different times. (E and F) The levels of inflammatory factors were detected by RT-qPCR in A549 cells transfected with pcDNA3.1-SOCS1 and lenti-crisprv2-SOCS1. Data represent means ± SEM. p values were determined by one-way ANOVA comparisons test. Asterisks denote the significance levels: $^{*}p < 0.05$, $^{**}p < 0.01$, and $^{***}p < 0.001$.

JAK/STAT pathway, thereby promoting inhibition of influenza virus replication. Overall, the presence of miR-19ab as a biomarker in patients with CHD has been linked to an increased susceptibility to influenza virus. Additionally, miR-19a/b has been identified as a potential target for the treatment of influenza and CHD.

### Conclusion

Patients with CHD are associated with downregulation of miR-19a/b, and this dysregulation may facilitate IAV infection. Overexpression of miR-19a/b reduces the mortality and prolongs the survival of CHD mice infected with H1N1. miR-19a/b can be considered as a preventative/therapy strategy for patients with CHD against influenza virus infection (Figure S8).

## MATERIALS AND METHODS

### Ethics statement and biosafety

All the animal experiments were conducted in accordance with the Guide for the Care and Use of Laboratory Animals published by the US National Institutes of Health, and the protocol was approved by the Committee on the Ethics of Animal Experiments of the Institute of Zoology, Chinese Academy of Sciences (approval number: IOZ-IACUC-2022-251). All experiments involving influenza viruses were performed in an Animal Biosafety Level 3 containment laboratory in the Research Center for Wildlife Diseases, which was approved by the Chinese Academy of Sciences.

### Cells and viruses

A549 cells (human airway epithelial cell line) and MDCK cells (Madin-Darby canine kidney cell line) were purchased from ATCC and cultured in DMEM (Dulbecco's modified Eagle medium; Gibco, London, UK) with 10% fetal bovine serum (Gibco) at 37°C in 5% $CO_2$. IAV BJ05/H1N1 (A/Beijing/05/2009(H1N1)) viruses were isolated and stored at the Institute of Zoology, Chinese Academy of Sciences. These viruses were propagated in 10-day-old specific pathogen-free (SPF) chicken embryos (Vital River Laboratories, Beijing, China). Virus titers determined for infection were calculated by plaque assay or 50% tissue culture infectious dose ($TCID_{50}$) titration on MDCK cells.

### Mice

$ApoE^{-/-}$ mice (males, ~8 weeks old, ~22 g bodyweight) with C57BL/6J background were purchased from Beijing SPF Laboratory Animal Technology. The animals were housed in SPF units of the Animal Center at Chinese Academy of Sciences, at 23°C ± 1°C, with a relative humidity of 60%–70% and a 12-h light/dark cycle. The animals can freely access to water and high-fat diet containing 41% fat plus 0.5% cholesterol (MD12015A, Medicience, Jiangsu, China) during the treatment. The animals were checked daily for food and water intake and body weight gain during the treatment. Studies were carried out under animal care guidelines of the Institute of Microbiology, Chinese Academy of Science, with license permit number SCXK20190010.

### Isolation of PBMC and total RNA was extracted from the cells

Whole blood was absorbed and thoroughly mixed with an equal amount of phosphate-buffered saline (PBS), 2 mL human lymphocyte separation solution was added, and the mixture was centrifuged at 2,000 rpm for 15min. PBMCs were absorbed and placed in 1.5 mL EP tube without enzyme; an appropriate amount of PBS was added and thoroughly mixed, then centrifuged; and precipitate was retained and stored at −80°C for testing. The protocol was approved by the Committee on the Ethics of Experiments of the Institute of Zoology, Chinese Academy of Sciences ( No. 2016PHB100-01 ) , and all patients gave informed consent.

### Agomir-RNA and miRNA transfection

micrON miRNA agomir-miR-19a, agomir-miR-19b, and agomir-NC were purchased from RiboBio (RiboBio, Guangdong, China). Agomir-miRNA and agomir-NC transfection was performed using the Lipofectamine 3000 transfection reagent (Thermo Fisher Scientific, Waltham, MA, USA) according to the manufacturer's instructions.

Sequences were as follows. hsa-miR-19a: agomir sense: 5′-AGUUU UGCAUAGUUGCACUACA-3′; antisense: 5′-AsCs GUGCAACUA UGCAAAA AsAsUsUs-Chol-3′. hsa-miR-19b: agomir sense: 5′-AG UUUUGCAGGUUUGCAUCCAGC-3′; antisense: 5′-AsCs GAUGC AAACCUGCAAAA AsAsUsUs-Chol-3′.

Normal saline was mixed with agomir-miR-19a, agomir-miR-19b, and agomir-NC. The mice that successfully established atherosclerosis models were randomly divided into an $ApoE^{-/-}$ mice group, $ApoE^{-/-}$+agomir-miR-19a/b-treated mice, and an agomir-NC mice group, with 10 mice in each group. The $ApoE^{-/-}$+agomir-miR-19a/b-treated mice group was injected with a mixture of 5 nmol agomir-miR-19a/b through the tail vein, the NC mice group was injected with a mixture of 5 nmol agomir-NC through the tail vein, and the $ApoE^{-/-}$ mice group was injected with the same amount of agomir-NC through the tail vein every 3 days for 3 weeks. RT-qPCR was used to detect whether miR-19a and miR-19b were overexpressed in $ApoE^{-/-}$+agomir-miR-19a/b-treated mice.

### miRNA quantification

Total RNA was isolated from infected and non-infected cells at different time points using Trizol Reagent (Thermo Fisher Scientific). Total RNA was treated with RNase-free DNase I (Roche, Indianapolis, IN, USA). Then, the total RNA was treated with poly(A) polymerase at 37°C for 1 h in a 20-μL-volume reaction mixture following the manufacturer's instruction. 1 μg total RNA was used to synthesize cDNA with a poly(T) adaptor. For each miRNA quantification, 50 ng cDNA was used in a volume of 25 μL mixture. The program was performed as follows: 95°C for 10 min, followed by 40 cycles of 95°C for 30 s, 55°C for 30 s, and 72°C for 30 s. The reactions were run on ABI7500 Fast (Applied Biosystems, Waltham, MA, USA)

### Virus infection

A549 cells were inoculated with BJ05/H1N1 virus at indicated titers in serum-free DMEM for 1 h at 37°C. The cells were washed three times with PBS and then cultured in 0.2 mL influenza virus growth medium, consisting of DMEM supplemented with 1% penicillin and streptomycin antibiotics, 0.2% bovine serum albumin (AMRESCO, Solon,

OH, USA), 25 mM HEPES (Life Technologies, Carlsbad, CA, USA), and 1 μg/mL TPCK-treated trypsin (Sigma-Aldrich, St. Louis, MO, USA) at 37°C in 5% $CO_2$.

#### Mouse infection

C57BL/6J mice, *ApoE*$^{-/-}$ mice, and *ApoE*$^{-/-}$+agomir-miR-19a/b-treated mice of the same age were challenged by intranasally inoculation. Briefly, mice were lightly anesthetized with $CO_2$ and intranasally inoculated with PBS and $10^5$ $TCID_{50}$ BJ/H1N1 influenza virus in a 100 μL volume. Body weight and clinical signs were recorded daily.

#### IAV quantification

Virus titrations were performed by $TCID_{50}$ assays. In brief, cell supernatants or grinding fluid of lung tissues were collected at indicated time points. One day prior to infection, MDCK cells were seeded in 96-well dishes with $3 \times 10^4$ cells in 0.2 mL DMEM plus 10% fetal bovine serum and 1% penicillin and streptomycin antibiotics. Confluent monolayer MDCK cells were inoculated with $^1/_2$ $\log_{10}$ serial dilutions of samples using cell media plus TPCK trypsin for dilutions. Cells were washed three times with PBS 1 h after being inoculated and cultured in 0.2 mL influenza virus growth medium. Three days after inoculation, cells were observed for endpoints in cytopathic effect or agglutination activity by using 1% (vol/vol) chicken red blood cells as an[10] indicator of virus replication in the cells. Virus titration was calculated by using the Reed-Muench method.

#### Pathological examination

Lungs were collected and fixed in 4% paraformaldehyde. Lung tissues were embedded in paraffin and cut into 5-μm sections. The sections were stained with H&E.

#### Immunofluorescence tissue staining

Lungs were collected and fixed in 4% paraformaldehyde. Lung tissues were embedded in paraffin and cut into 5-μm sections. Briefly, the sections were deparaffinized with serial ethanol and dealt with antigen retrieval buffer preceding permeabilization with 0.5% Trition-100. Virus antigen was stained using a monoclonal antibody of H1N1 influenza virus NP protein and visualized with a secondary goat anti-rabbit antibody conjugated to fluorescein isothiocyanate. Sections were also stained for DNA with DAPI (Sigma).

#### Cytokine or chemokine analysis

Lungs were collected and homogenized on days 1, 2, 3, 5, and 7 post-infection. Total RNA samples were extracted using Trizol Reagent. Briefly, TNF-α, IFN-γ, IL-1β, IL-4, IL-6, and MIP-1α were quantified using SYBR I-based real-time PCR. The results were normalized to GAPDH and WT mice on day 0 post-infection. The primers used in this assay are listed in Figure S10.

#### Western blot

The proteins were extracted from the lung tissues using RIPA lysis (Beyotime, Shanghai, China) and then measured by BCA kit (Beyotime) to determine protein concentration. After that, the proteins were mixed with loading buffer in a boiled water bath for 3 min for denature. Then, the proteins were subjected to electrophoresis at 80 V for 30 min and then 120 V for 1–2 h. The proteins were transferred into the membrane on ice bath at 300 mA for 60 min. The membranes were blocked in confining liquid for 60 min or 4°C overnight before being incubated into primary antibody for β-actin (44035, 1:1,000), NP (125989, 1:1,000), M1 (127356, 1:1,000), SOCS1 (37038, 1:1,000), JAK1 (bs-1439R, 1:1,000), pSTAT1 (ab30645, 1:1,000), and STAT1 (ab31369, 1:1,000) (all from Cell Signaling, Boston, MA, USA) for 1 h. After incubation, the membranes were washed in washing buffer for 3 × 10 min before secondary antibody was applied for incubation at room temperature for 1 h. After being washed for 3 × 10 min, the membranes were added with developer solution for color development. The color development was verified in a chemiluminescent imaging system (Bio-Rad, Hercules, CA, USA).

#### Statistical analysis

Statistical analysis was performed using GraphPad Prism 8.0.2. Statistical significance was determined by two-tailed unpaired Student's t test or by one-way/two-way ANOVA, and p values <0.05 are considered significant and denoted as $^*p < 0.05$, $^{**}p < 0.01$, and $^{***}p < 0.001$. Non-significant values are denoted as ns.

## DATA AND CODE AVAILABILITY

The data presented in the study are deposited in the National Genomics Data Center, accession no. PRJCA018256.

## SUPPLEMENTAL INFORMATION

Supplemental information can be found online at https://doi.org/10.1016/j.omtn.2024.102149.

## ACKNOWLEDGMENTS

This study was supported by the National Key R&D Program of China (2022YFC2601602), the National Forestry and Grassland Administration, and the China and Beijing Wildlife Rescue Center, China. We acknowledge BioRender, which was used to create the graphical abstract.

## AUTHOR CONTRIBUTIONS

H.H. and Y.X. contributed to the conception of the study; L.C. contributed significantly to collecting samples and performing the experiment; B.H., Y.L., G.L., S.H., Y.W., and Y.H. contributed significantly to analysis and manuscript preparation; Y.T., W.Z., H.M., and Y.G. helped perform the analysis with constructive discussions; and Y.X. performed the data analyses and wrote the manuscript.

## DECLARATION OF INTERESTS

The authors declare no competing interests.

## REFERENCES

1. Si, L., Xu, H., Zhou, X., Zhang, Z., Tian, Z., Wang, Y., Wu, Y., Zhang, B., Niu, Z., Zhang, C., et al. (2016). Generation of influenza A viruses as live but replication-incompetent virus vaccines. Science *354*, 1170–1173.

2. Kosik, I., Ince, W.L., Gentles, L.E., Oler, A.J., Kosikova, M., Angel, M., Magadán, J.G., Xie, H., Brooke, C.B., and Yewdell, J.W. (2018). Influenza A virus hemagglutinin

glycosylation compensates for antibody escape fitness costs. PLoS Pathog. 14, e1006796.

3. Pan, Y., Cui, S., Sun, Y., Zhang, X., Ma, C., Shi, W., Peng, X., Lu, G., Zhang, D., Liu, Y., et al. (2018). Human infection with H9N2 avian influenza in northern China. Clin. Microbiol. Infect. 24, 321–323.
4. Tumpey, T.M., Basler, C.F., Aguilar, P.V., Zeng, H., Solórzano, A., Swayne, D.E., Cox, N.J., Katz, J.M., Taubenberger, J.K., Palese, P., and García-Sastre, A. (2005). Characterization of the reconstructed 1918 Spanish influenza pandemic virus. Science 310, 77–80.
5. Louie, J.K., Jean, C., Acosta, M., Samuel, M.C., Matyas, B.T., and Schechter, R. (2011). A review of adult mortality due to 2009 pandemic (H1N1) influenza A in California. PLoS One 6, e18221.
6. Writing Committee of the WHO Consultation on Clinical Aspects of Pandemic H1N1 2009 Influenza, Bautista, E., Chotpitayasunondh, T., Gao, Z., Harper, S.A., Shaw, M., Uyeki, T.M., Zaki, S.R., Hayden, F.G., Hui, D.S., et al. (2010). Clinical aspects of pandemic 2009 influenza A (H1N1) virus infection. N. Engl. J. Med. 362, 1708–1719.
7. Dawood, F.S., Chung, J.R., Kim, S.S., Zimmerman, R.K., Nowalk, M.P., Jackson, M.L., Jackson, L.A., Monto, A.S., Martin, E.T., Belongia, E.A., et al. (2020). Interim Estimates of 2019-20 Seasonal Influenza Vaccine Effectiveness - United States, February 2020. MMWR Morb. Mortal. Wkly. Rep. 69, 177–182.
8. Azambuja, M.I.R., and Duncan, B.B. (2002). Similarities in mortality patterns from influenza in the first half of the 20th century and the rise and fall of ischemic heart disease in the United States: a new hypothesis concerning the coronary heart disease epidemic. Cad. Saúde Pública 18, 557–577. discussion 67-77.
9. Chiong, M., Wang, Z.V., Pedrozo, Z., Cao, D.J., Troncoso, R., Ibacache, M., Criollo, A., Nemchenko, A., Hill, J.A., and Lavandero, S. (2011). Cardiomyocyte death: mechanisms and translational implications. Cell Death Dis. 2, e244.
10. Pedicino, D., Giglio, A.F., Galiffa, V.A., Cialdella, P., Trotta, F., Graziani, F., and Liuzzo, G. (2013). Infections, immunity and atherosclerosis: pathogenic mechanisms and unsolved questions. Int. J. Cardiol. 166, 572–583.
11. Naghavi, M., Wyde, P., Litovsky, S., Madjid, M., Akhtar, A., Naguib, S., Siadaty, M.S., Sanati, S., and Casscells, W. (2003). Influenza infection exerts prominent inflammatory and thrombotic effects on the atherosclerotic plaques of apolipoprotein E-deficient mice. Circulation 107, 762–768.
12. Mahalle, N., Kulkarni, M.V., Garg, M.K., and Naik, S.S. (2013). Vitamin B12 deficiency and hyperhomocysteinemia as correlates of cardiovascular risk factors in Indian subjects with coronary artery disease. J. Cardiol. 61, 289–294.
13. Gallaher, A.M., Das, S., Xiao, Z., Andresson, T., Kieffer-Kwon, P., Happel, C., and Ziegelbauer, J. (2013). Proteomic Screening of Human Targets of Viral microRNAs Reveals Functions Associated with Immune Evasion and Angiogenesis. PLoS Pathog. 9, e1003584.
14. Trobaugh, D.W., Gardner, C.L., Sun, C., Haddow, A.D., Wang, E., Chapnik, E., Mildner, A., Weaver, S.C., Ryman, K.D., and Klimstra, W.B. (2014). RNA viruses can hijack vertebrate microRNAs to suppress innate immunity. Nature 506, 245–248.
15. Bartel, D.P. (2004). MicroRNAs: Genomics, biogenesis, mechanism, and function. Cell 116, 281–297.
16. Cullen, B.R. (2013). How do viruses avoid inhibition by endogenous cellular microRNAs? PLoS Pathog. 9, e1003694.
17. Wienholds, E., and Plasterk, R.H.A. (2005). MicroRNA function in animal development. FEBS Lett. 579, 5911–5922.
18. Ma, Y.J., Yang, J., Fan, X.L., Zhao, H.B., Hu, W., Li, Z.P., Yu, G.C., Ding, X.R., Wang, J.Z., Bo, X.C., et al. (2012). Cellular microRNA let-7c inhibits M1 protein expression of the H1N1 influenza A virus in infected human lung epithelial cells. J. Cell Mol. Med. 16, 2539–2546.
19. Tambyah, P.A., Sepramaniam, S., Mohamed Ali, J., Chai, S.C., Swaminathan, P., Armugam, A., and Jeyaseelan, K. (2013). microRNAs in circulation are altered in response to influenza A virus infection in humans. PLoS One 8, e76811.
20. Hong, L., Lai, M., Chen, M., Xie, C., Liao, R., Kang, Y.J., Xiao, C., Hu, W.Y., Han, J., and Sun, P. (2010). The miR-17-92 cluster of microRNAs confers tumorigenicity by inhibiting oncogene-induced senescence. Cancer Res. 70, 8547–8557.
21. Han, Y.C., Vidigal, J.A., Mu, P., Yao, E., Singh, I., Gonzalez, A.J., Concepcion, C.P., Bonetti, C., Ogrodowski, P., Carver, B., et al. (2015). An allelic series of miR-17 similar to 92-mutant mice uncovers functional specialization and cooperation among members of a microRNA polycistron. Nat. Genet. 47, 766.
22. Mayer, O., Jr., Seidlerová, J., Černá, V., Kučerová, A., Vaněk, J., Karnosová, P., Bruthans, J., Wohlfahrt, P., Cífková, R., Pešta, M., and Filipovský, J. (2019). The low expression of circulating microRNA-19a represents an additional mortality risk in stable patients with vascular disease. Int. J. Cardiol. 289, 101–106.
23. Singh, S., de Ronde, M.W.J., Creemers, E.E., Van der Made, I., Meijering, R., Chan, M.Y., Hwee Tan, S., Tang Chin, C., Mark Richards, A., Troughton, R.W., et al. (2021). Low miR-19b-1-5p Expression Is Related to Aspirin Resistance and Major Adverse Cardio- Cerebrovascular Events in Patients With Acute Coronary Syndrome. J. Am. Heart Assoc. 10, e017120.
24. Gao, F., Kataoka, M., Liu, N., Liang, T., Huang, Z.P., Gu, F., Ding, J., Liu, J., Zhang, F., Ma, Q., et al. (2019). Therapeutic role of miR-19a/19b in cardiac regeneration and protection from myocardial infarction. Nat. Commun. 10, 1802.
25. Lin, X., Yu, S., Ren, P., Sun, X., and Jin, M. (2020). Human microRNA-30 inhibits influenza virus infection by suppressing the expression of SOCS1, SOCS3, and NEDD4. Cell Microbiol. 22, e13150.
26. Zhuang, Y., Li, T., Xiao, H., Wu, J., Su, S., Dong, X., Hu, X., Hua, Q., Liu, J., Shang, W., et al. (2021). LncRNA-H19 Drives Cardiomyocyte Senescence by Targeting miR-19a/socs1/p53 Axis. Front. Pharmacol. 12, 631835.
27. Baker, B.J., Akhtar, L.N., and Benveniste, E.N. (2009). SOCS1 and SOCS3 in the control of CNS immunity. Trends Immunol. 30, 392–400.
28. Yang, C., Jia, X., Zhou, J., Sun, Q., and Ma, Z. (2020). The MiR-17-92 Gene Cluster is a Blood-Based Marker for Cancer Detection in Non-Small-Cell Lung Cancer. Am. J. Med. Sci. 360, 248–260.
29. Mogilyansky, E., and Rigoutsos, I. (2013). The miR-17/92 cluster: a comprehensive update on its genomics, genetics, functions and increasingly important and numerous roles in health and disease. Cell Death Differ. 20, 1603–1614.
30. Sakai, A., Saitow, F., Maruyama, M., Miyake, N., Miyake, K., Shimada, T., Okada, T., and Suzuki, H. (2017). MicroRNA cluster miR-17-92 regulates multiple functionally related voltage-gated potassium channels in chronic neuropathic pain. Nat. Commun. 8, 16079.
31. Shimakami, T., Yamane, D., Jangra, R.K., Kempf, B.J., Spaniel, C., Barton, D.J., and Lemon, S.M. (2012). Stabilization of hepatitis C virus RNA by an Ago2-miR-122 complex. Proc. Natl. Acad. Sci. USA 109, 941–946.
32. Lanford, R.E., Hildebrandt-Eriksen, E.S., Petri, A., Persson, R., Lindow, M., Munk, M.E., Kauppinen, S., and Ørum, H. (2010). Therapeutic silencing of microRNA-122 in primates with chronic hepatitis C virus infection. Science 327, 198–201.
33. Elliott, J., and Johnston, J.A. (2004). SOCS: role in inflammation, allergy and homeostasis. Trends Immunol. 25, 434–440.
34. Dalpke, A.H., Opper, S., Zimmermann, S., and Heeg, K. (2001). Suppressors of cytokine signaling (SOCS)-1 and SOCS-3 are induced by CpG-DNA and modulate cytokine responses in APCs. J. Immunol. 166, 7082–7089.
35. Bartel, D.P. (2009). MicroRNAs: target recognition and regulatory functions. Cell 136, 215–233.
36. Dang, L., Teng, M., Li, H.Z., Ma, S.M., Lu, Q.X., Hao, H.F., Zhao, D., Zhou, E.M., Zhang, G.P., and Luo, J. (2017). Marek's disease virus type 1 encoded analog of miR-155 promotes proliferation of chicken embryo fibroblast and DF-1 cells by targeting hnRNPAB. Vet. Microbiol. 207, 210–218.
37. Deng, Y., Yan, Y., Tan, K.S., Liu, J., Chow, V.T., Tao, Z.Z., and Wang, D.Y. (2017). MicroRNA-146a induction during influenza H3N2 virus infection targets and regulates TRAF6 levels in human nasal epithelial cells (hNECs). Exp. Cell Res. 352, 184–192.
38. Chang, J., Guo, J.T., Jiang, D., Guo, H., Taylor, J.M., and Block, T.M. (2008). Liver-specific microRNA miR-122 enhances the replication of hepatitis C virus in nonhepatic cells. J. Virol. 82, 8215–8223.
39. Zhou, Y., Liu, Y., Yan, H., Li, Y., Zhang, H., Xu, J., Puthiyakunnon, S., and Chen, X. (2014). miR-281, an abundant midgut-specific miRNA of the vector mosquito Aedes albopictus enhances dengue virus replication. Parasites Vectors 7, 488.

**OMTN, Volume *35***

## Supplemental information

# Therapeutic role of miR-19a/b protection from influenza virus infection in patients with coronary heart disease

**Yanan Xing, Lin Chen, Bin Hu, Yi Li, Huan Mai, Gaojian Li, Shuyi Han, Ye Wang, Yanyi Huang, Ying Tian, Wei Zhang, Yan Gao, and Hongxuan He**

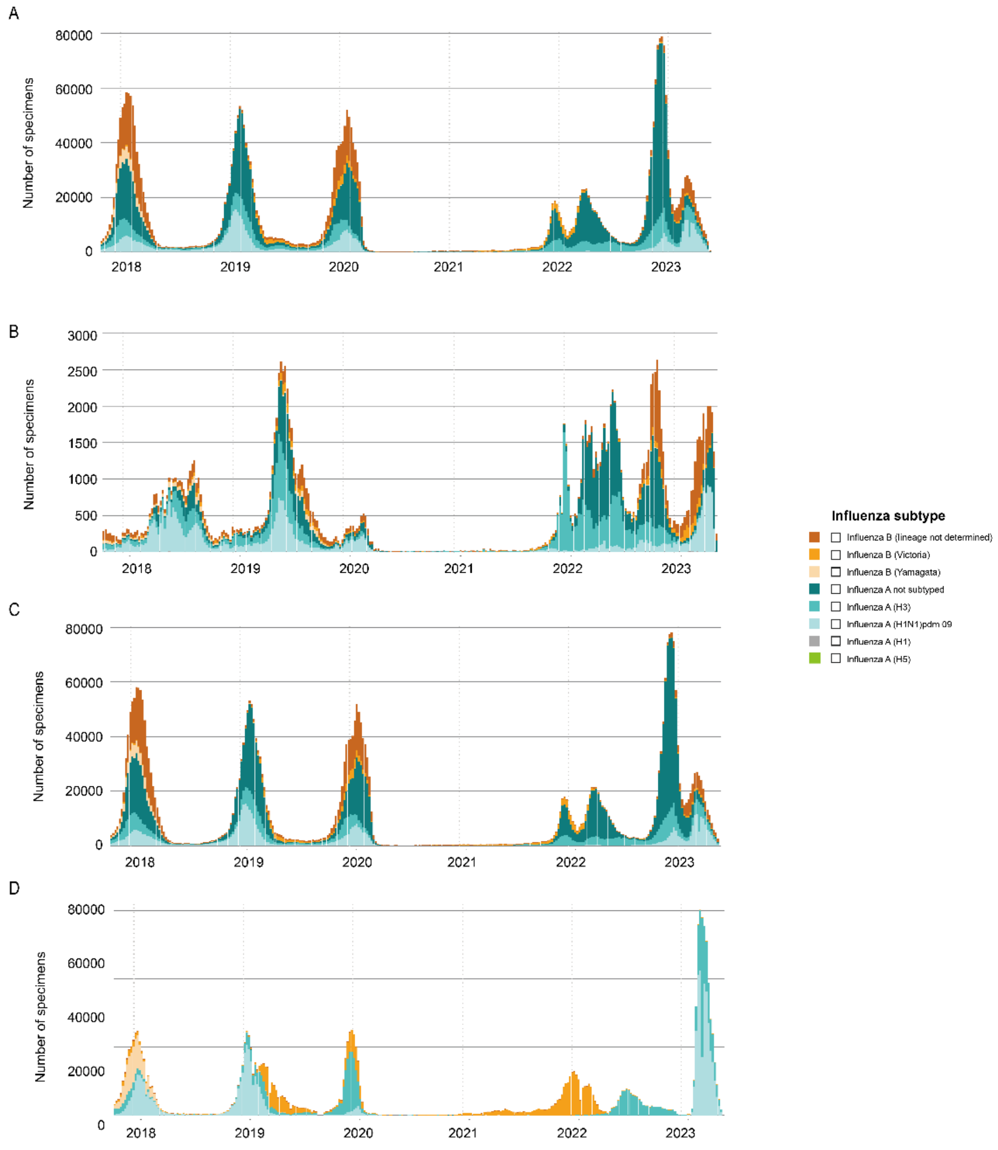


**Figure S1** Patterns of influenza virus circulation in the global world、Northern (top) 、Southern (bottom) Hemispheres and China, Jan 2018 to present. Data are based on laboratory confirmed influenza activity reported to WHO flunet. https://www.who.int/tools/flunet

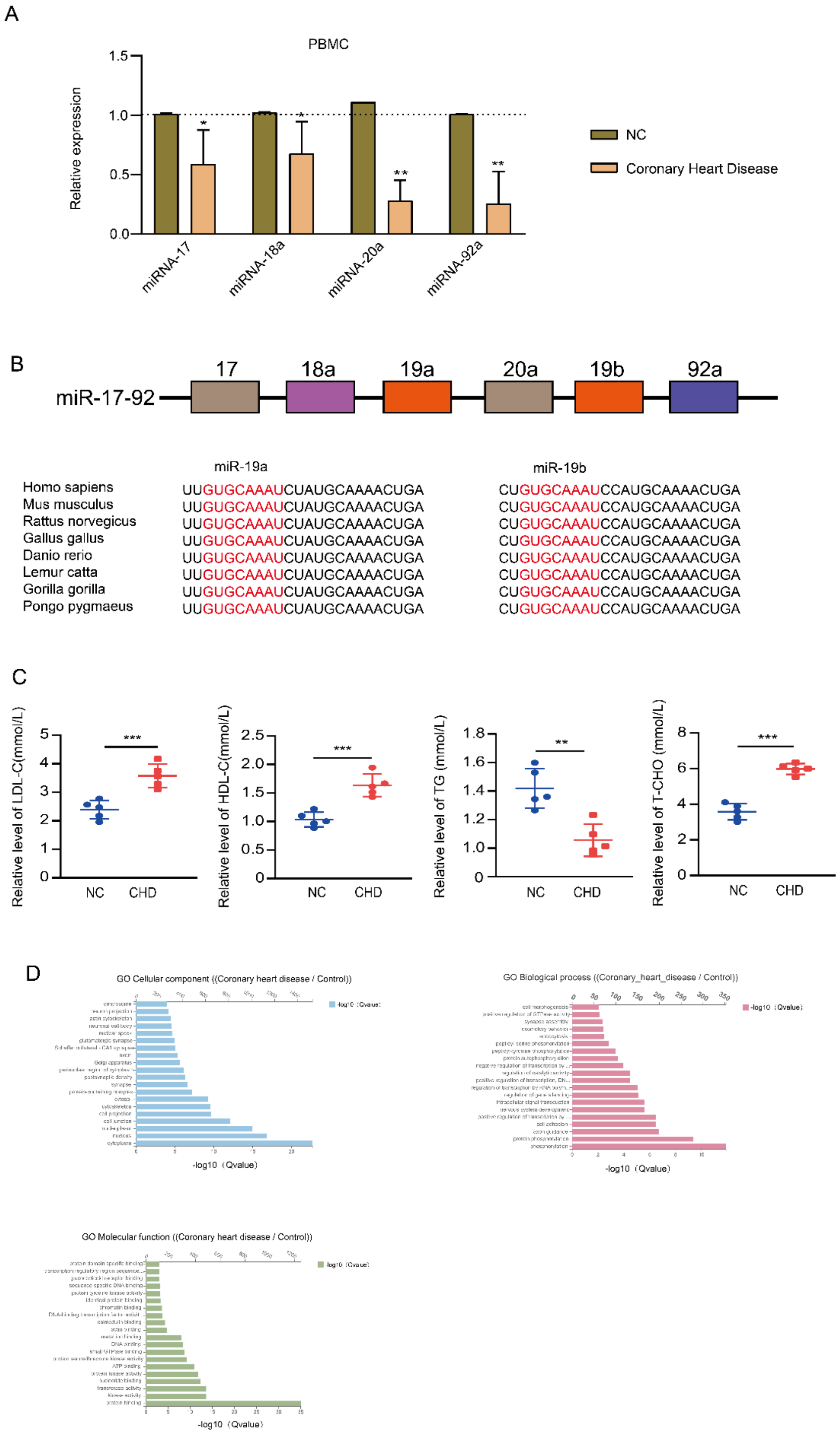
A
PBMC
Relative expression
1.5
1.0
0.5
0.0
*
*
**
**
miRNA-17
miRNA-18a
miRNA-20a
miRNA-92a
NC
Coronary Heart Disease
B
miR-17-92
17
18a
19a
20a
19b
92a
miR-19a
miR-19b
Homo sapiens UUGUGCAAAUCUAUGCAAAACUGA CUGUGCAAAUCCAUGCAAAACUGA
Mus musculus UUGUGCAAAUCUAUGCAAAACUGA CUGUGCAAAUCCAUGCAAAACUGA
Rattus norvegicus UUGUGCAAAUCUAUGCAAAACUGA CUGUGCAAAUCCAUGCAAAACUGA
Gallus gallus UUGUGCAAAUCUAUGCAAAACUGA CUGUGCAAAUCCAUGCAAAACUGA
Danio rerio UUGUGCAAAUCUAUGCAAAACUGA CUGUGCAAAUCCAUGCAAAACUGA
Lemur catta UUGUGCAAAUCUAUGCAAAACUGA CUGUGCAAAUCCAUGCAAAACUGA
Gorilla gorilla UUGUGCAAAUCUAUGCAAAACUGA CUGUGCAAAUCCAUGCAAAACUGA
Pongo pygmaeus UUGUGCAAAUCUAUGCAAAACUGA CUGUGCAAAUCCAUGCAAAACUGA
C
Relative level of LDL-C(mmol/L)
***
NC
CHD
Relative level of HDL-C(mmol/L)
***
NC
CHD
Relative level of TG (mmol/L)
**
NC
CHD
Relative level of T-CHO (mmol/L)
***
NC
CHD
D
GO Cellular component ((Coronary heart disease / Control))
-log10（Qvalue）
GO Biological process ((Coronary_heart_disease / Control))
-log10（Qvalue）
GO Molecular function ((Coronary heart disease / Control))
-log10（Qvalue）

**Figure S2.** A. Expression of miR-17, miR-18a, miR-20a and miR-92a detected by qRT-PCR in PBMC of CHD patients and healthy people. B. Biochemical indicators of CHD patients and healthy people. C. Statistics of GO entries enriched with differential microRNAs were enriched between CHD mice and normal mice. Note: BP: BP enrichment, cellular component (CC): CC enrichment, and molecular function (MF): MF enrichment.

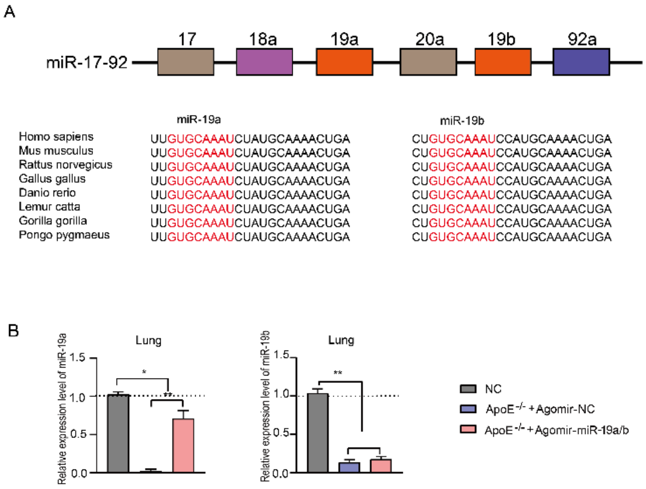


**Figure S3. A.** Gene structure of the miR-17–92 cluster and conserved sequences of miR-19a and miR-19b across species. Seed sequences are highlighted. B. Expression of miR-19a detected by qRT-PCR in lungs of NC mice, ApoE-/- + agomir-NC mice, and ApoE-/- + agomir-miR-19a/b-treated mice.

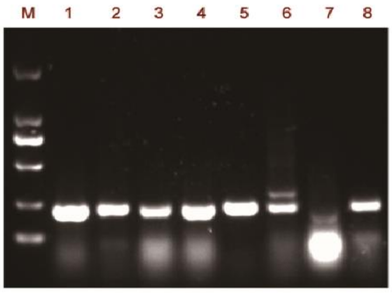


**Figure S4.** M represents DL2000 marker; lanes 1to 6 represents lung, heart, liver, spleen, kidney, brain, lane 7: Negative control; lane 8: Positive control;

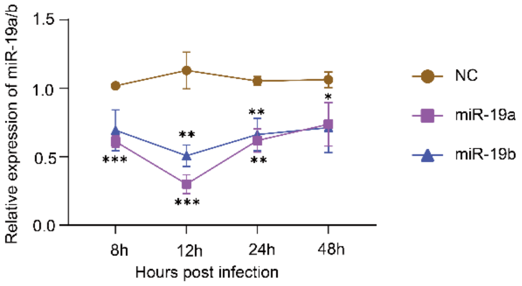


**Figure S5.** the expression of miR-19a/b was time- and dose-dependent on NDV virus infection detected by quantitative analysis. Asterisks denote the significance levels: * $p<0.05$; ** $p<0.01$; *** $p<0.001$.

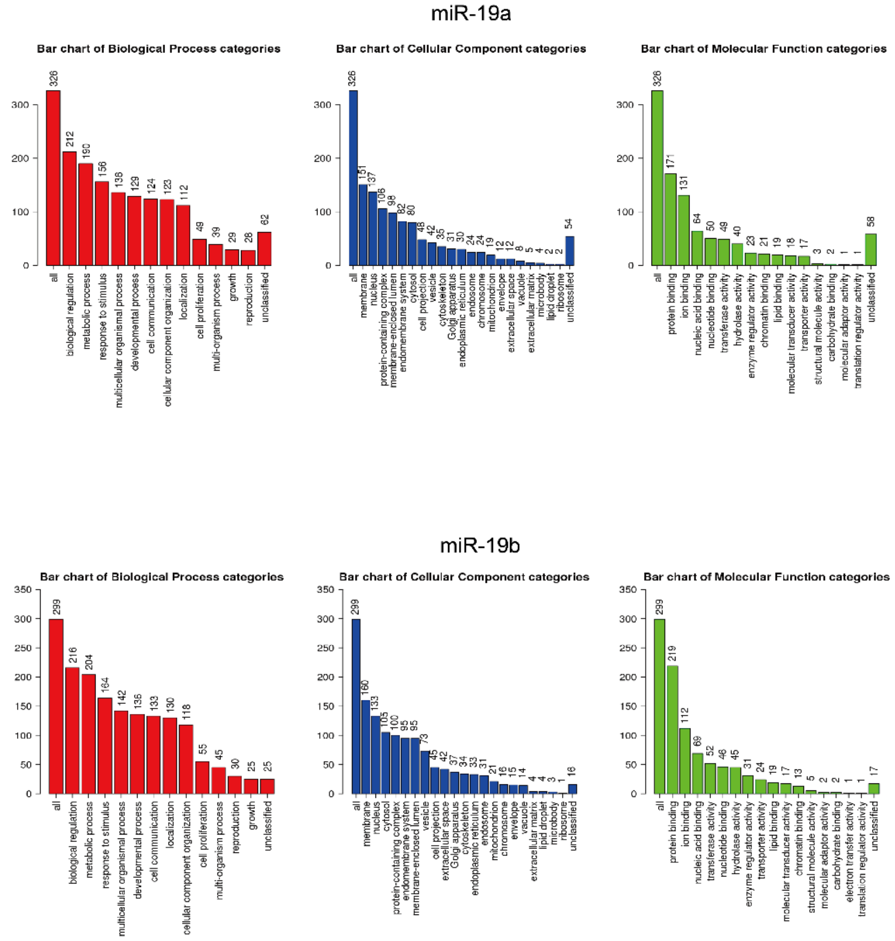


**Figure S6.** Statistics of GO entries enriched with miR-19a and miR-19b target genes.

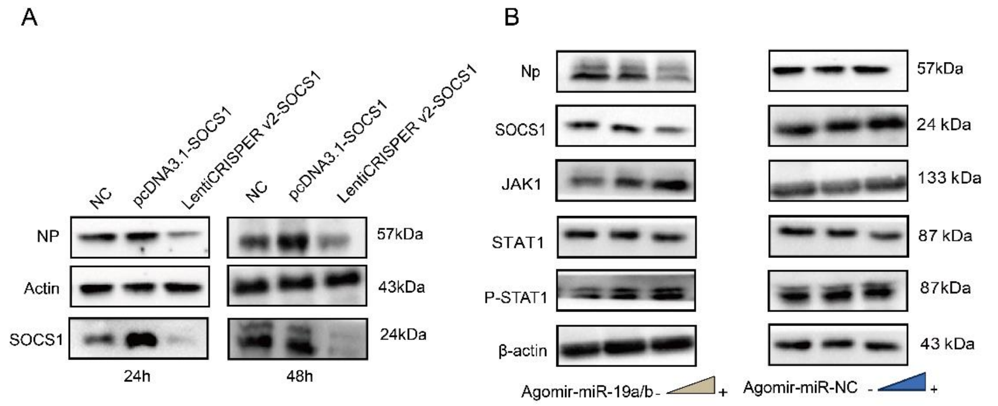


**Figure S7.A.** A549 cells were transfected with pcDNA3.1-SOCS1, lenti-crisprv2-SOCS1 and empty vector for 48 hours, and then infected with BJ05/H1N1 24h.p.i. western blot detection of NP level in A549 cells were transfected with pcDNA3.1-SOCS1, lenti-crisprv2-SOCS1 and empty vector in different time. B. A549 cells were transfected with agomir-miR-19a/b and agomir-NC at different concentrations and after infected with BJ05/H1N1 24h.p.i，then detected NP, M1, SOCS1, JAK2, STAT1 and p-STAT1 expression levels were analyzed using Western blot.

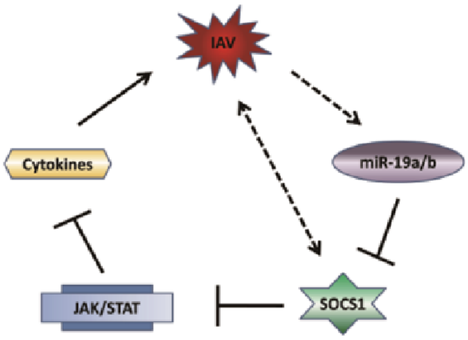


**Figure S8.** C. A hypothetical model for the role of miR-19/SOCS1 axis in IAV induced inflammatory cytokine expression. Solid arrows, signaling pathways identified in this study; broken arrows, potential signaling pathways.

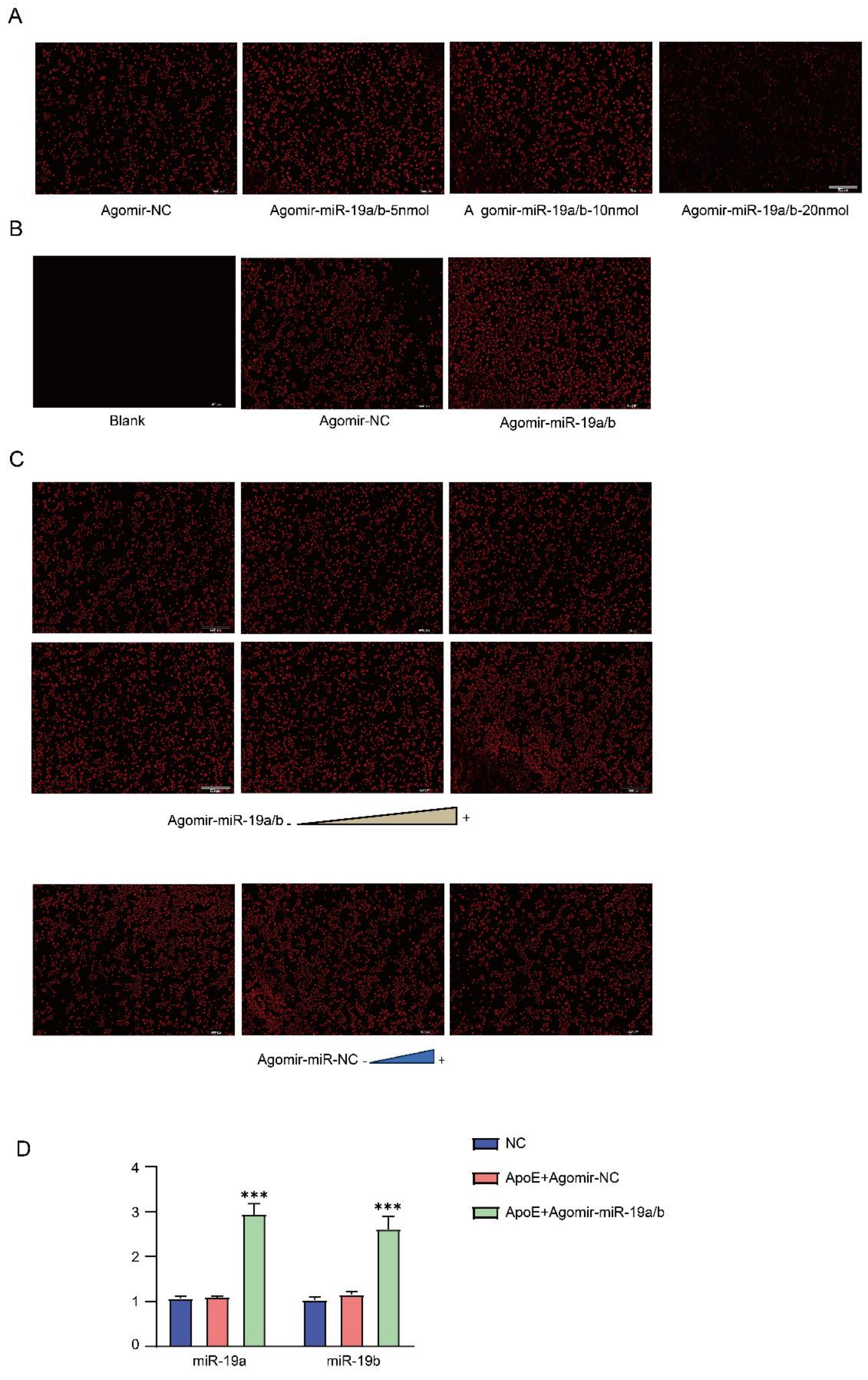


**Figure S9.** A. Transfection efficiency of Fig.4A. B. Transfection efficiency of Fig.4B. C. Transfection efficiency of Fig. 6C. D. Transfection efficiency of Fig.5D.

| Targets | Primer sequence |
|---|---|
| β-actin F | CACCAGTTCGCCATGGAT |
| β-actin R | CCTCGTCACCCACATAGGAG |
| SOCS1 F | TTGGAGGGAGCGGATGGGTGTAG |
| SOCS1 R | AGAGGTAGGAGGTGCGAGTTCAGGTC |
| IFN-α F | GAAGGACAGGAAGGATTTGGA |
| IFN-α R | TGAGCCTTCTGGBATCTGTTGGT |
| TNF-α F | CATCTTCTCAAAATTCGAGTGACAA |
| TNF-α R | TGGGAGTAGACAAGGTACAACCC |
| IL-β F | CAACCAACAAGTGATATTCTCCATG |
| IL-β R | GATCCACACTCTCCAGCTGCA |
| IL-6 F | GAGGATACCACTCCCAACAGACC |
| IL-6 R | AAGTGCATCATCGTTGTTCATACA |
| U6 F | CTCGCTTCGGCAGCACATATACT |
| U6 R | CGCTTCACGAATTTGCGTGT |
| GAPDH F | RCTCAGTGTAGCCCAGGATGC |
| GAPDH R | FACCACCATGGAGAAGGCTGG |
| SOCS1WT F | GGATCTTCCAGAGATGAGCTCTGTGGG TACCCTCCCCGG |
| SOCS1WT R | CTGCCGTTCGACGATCTCGAGAAAAAA AAAACTTTCATAATAAAGTTTATTACC |
| SOCS1-MUT F | TCAAACGTGTAACCAGGGGTTGGGGG AGGGTCT |
| SOCS1-MUT R | CCCTGGTTACACGTTTGATACTGGGTAT ATGTAAACATGAAGAGG |

**Figure S10. Primer sequence**
